# Cost-effectiveness of health technologies in adults with type 1 diabetes: a systematic review and narrative synthesis

**DOI:** 10.1186/s13643-020-01373-y

**Published:** 2020-08-03

**Authors:** Anthony Pease, Ella Zomer, Danny Liew, Clement Lo, Arul Earnest, Sophia Zoungas

**Affiliations:** 1 School of Public Health and Preventive Medicine, Monash University, 553 St Kilda Road, Melbourne, Victoria 3004 Australia; 2 Monash Health, Melbourne, Victoria Australia; 3 Alfred Health, Melbourne, Victoria Australia

**Keywords:** Economics or medical economics, Type 1 diabetes, Insulin pumps, Continuous glucose monitoring, Closed-loop systems, Flash glucose monitoring, Narrative synthesis

## Abstract

**Background:**

With the rapid development of technologies for type 1 diabetes, economic evaluations are integral in guiding cost-effective clinical and policy decisions. We therefore aimed to review and synthesise the current economic literature for available diabetes management technologies and outline key determinants of cost-effectiveness.

**Methods:**

A systematic search was conducted in April 2019 that focused on modelling or trial based economic evaluations. Searched databases included Medline, Medline in-process and other non-indexed citations, EMBASE, PubMed, All Evidenced Based Medicine Reviews, EconLit, Cost-effectiveness analysis Registry, Research Papers in Economics, Web of Science, PsycInfo, CINAHL, and PROSPERO from inception. We assessed quality of included studies with the Questionnaire to Assess Relevance and Credibility of Modeling Studies for Informing Health Care Decision Making an ISPOR-AMCP-NPC good practice task force report. Screening of abstracts and full-texts, appraisal, and extraction were performed by two independent researches.

**Results:**

We identified 16,772 publications, of which 35 were analysed and included 11 health technologies. Despite a lack of consensus, most studies reported that insulin pumps (56%) or interstitial glucose sensors (62%) were cost-effective, although incremental cost-effectiveness ratios ranged widely ($14,266–$2,997,832 USD). Cost-effectiveness for combined insulin pumps and glucose sensors was less clear. Determinants of cost-effectiveness included treatment effects on glycosylated haemoglobin and hypoglycaemia, costing of technologies and complications, and measures of utility.

**Conclusions:**

Insulin pumps or glucose sensors appeared cost-effective, particularly in populations with higher HbA1c levels and rates of hypoglycaemia. However, cost-effectiveness for combined insulin pumps and glucose sensors was less clear.

**Registration:**

The study was registered with PROSPERO, number CRD42017077221.

## Background

Millions of adults are estimated to be living with type 1 diabetes and are dependent on exogenous insulin to regulate blood glucose levels [1, 2]. The complications of dysglycaemia in this population contribute to disproportionately high morbidity, mortality, and healthcare expenses [1–3].

Increasingly advanced technologies have been developed over the last 40 to 50 years to improve glycaemia and prevent complications of diabetes [4, 5]. The uptake of these devices for insulin delivery, glucose sensing, and insulin-dose advice is also growing internationally [6–9]. However, new technologies are infrequently compared with the full range of alternatives in terms of clinical outcomes or cost-effectiveness. Furthermore, funding options for these management devices in some settings may prevent patients from accessing treatment regardless of the potential clinical benefit [10–12].

Given the rapid development of technologies, it is critical for economic evaluations of all technologies to be synthesised to guide cost-effective device selection. There have been few systematic reviews of economic evaluations to date, and these become increasingly outdated as new devices become available and healthcare funding changes. The 2015 systematic review of continuous subcutaneous insulin infusion (CSII) pumps concluded this form of insulin delivery was cost-effective compared to multiple daily injections (MDI) [13]. Another 2015 systematic review of continuous glucose monitoring (CGM) concluded that continuous glucose sensing technology was not cost-effective when compared to self-monitoring of capillary blood glucose (SMBG) based on two included studies [14]. In contrast, the individual studies reported that CGM was cost-effective although with significant uncertainty [15, 16]. A systematic review from 2016 included two studies regarding the combination of CSII with CGM technology but drew no conclusions apart from review authors’ own separate economic evaluation [17]. There has been a large number of economic studies since these reviews were published, and to our knowledge, no other review has synthesised economic evaluations for multiple technologies.

Therefore, we provide a systematic review and narrative synthesis of economic outcomes for diabetes management technologies among adults with type 1 diabetes, following network geometry analogous to network meta-analyses. We aimed to summarise currently available economic evaluations for insulin delivery, glucose sensing, and decision support technologies, and identify factors conducive to cost-effectiveness in order to guide clinician and care provider decisions.

## Methods

### Search strategy and selection criteria

We performed a systematic review and narrative synthesis. We searched Medline, Medline in-process and other non-indexed citations, EMBASE, PubMed, All Evidenced Based Medicine Reviews, EconLit, Cost-effectiveness analysis Registry, Research Papers in Economics, Web of Science, PsycInfo, CINAHL, and PROSPERO from the date of their inception to 24 April 2019, limited to the English language.

We included full and partial economic evaluations that were based on modelling or randomised controlled trials (RCTs) of parallel and crossover study design, six or more weeks in duration, and included community dwelling adults (>18 years of age) with type 1 diabetes. Pregnancy was an exclusion. Because HbA1c reflects the preceding weeks to months of glycaemia, expert consensus opinion deemed 6 weeks to be the minimum study duration that would reasonably allow interpretation of treatment effects for interventions. Very few economic evaluations modelled an entirely adult cohort with all input parameters also based on studies limited to adult participants. We therefore excluded studies if the modelled cohort was limited solely to paediatric participants (< 18 years of age). We considered studies that compared technologies for insulin delivery, glucose monitoring, insulin dosing advice, or multiple daily injections (MDI) and self-monitoring of blood glucose via capillary testing (SMBG). Because every individual with type 1 diabetes must have at least one method for insulin delivery and glucose monitoring, we considered 11 intervention-pairs based on the results of our searches. The electronic database searches were supplemented by manual searches of reference lists of review articles.

The lead investigator (AP) screened titles, abstracts, and full text articles where appropriate with independent duplication for a convenience sample (CL; 94% screened with 100% agreement). Three investigators (AP, EZ, GD) reviewed the main reports and supplementary materials, and assessed the relevance and credibility for all eligible studies. Two investigators (AP, EZ) extracted relevant summary estimates for economic outcomes. Any discrepancies were resolved by consensus or deferral to a senior reviewer (SZ or DL). The protocol of this analysis has been published (https://www.ncbi.nlm.nih.gov/pubmed/29530081) [18] and the review was written in accordance with the Preferred Reporting Items for Systematic Reviews and Meta-Analyses (PRISMA) statement guideline [19]. The PRISMA checklist was completed (Additional file 1).

### Outcomes

The primary outcomes of interest were costs and cost-effectiveness. Clinical outcomes from our review protocol have been reported separately [20]. We extracted data regarding costs, quality adjusted life years (QALYs), and incremental cost effectiveness ratios (ICERs), as well as cost-calculations, patient time-costs, and budget impact analysis. For comparison, we also extracted published or assumed willingness-to-pay thresholds, and converted ICERs to 2019 Australian Dollars (AUD) and United States Dollars (USD) using relevant exchange rates from https://www.oanda.com/currency/converter/ [21]; and inflation rates (average consumer price index) from the International Monetary Fund World Economic Outlook Database [22].

### Data analysis

We assessed the studies’ relevance and credibility by following the good practice task force report by the International Society for Pharmacoeconomics and Outcomes Research (ISPOR), Academy of Managed Care Pharmacy (AMCP), and the National Pharmaceutical Council (NPC) [23]. Because there is no consensus on methods to pool cost-effectiveness estimates, meta-analysis was not possible and narrative synthesis was performed. Unless otherwise stated, results were reported as 2019 Australian (United States) dollars.

### Registration

This study was registered with PROSPERO, number CRD42017077221.

## Results

**Fig. 1:**
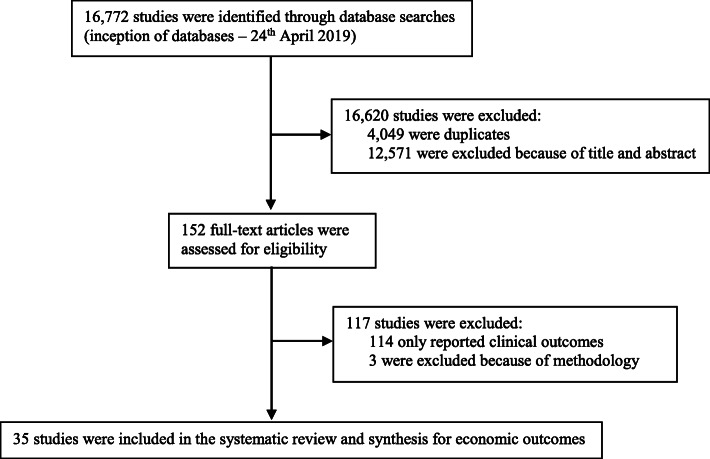
Study selection: Article counts through the systematic review process

We identified 16,772 records, of which 152 potentially eligible publications were retrieved in full text (Fig. 1). Of these, 114 reported clinical outcomes only; three economic evaluations were not based on modelling or RCTs, leaving 35 full-text articles for analysis (Additional file 2).

Summary findings and review of the 30 cost-effectiveness analyses, two cost-calculations, two budget impact models, one patient time-cost analysis, and one real option analysis among patients with type 1 diabetes were completed (Additional file 3 and 4) and narrative synthesis performed.

The quality assessment deemed all studies to be of adequate relevance to the review, and to have adequate credibility. The study by Herman et al. [24] had limited generalisability due to the comparator arm comprising MDI and only one capillary blood glucose test per day, which is not a recommended treatment strategy for type 1 diabetes. The study by Haahtela [25] comparing integrated systems with low glucose suspend to MDI with SMBG did not trigger a ‘fatal flaw’ rating for credibility but had multiple neutral ratings because adequate details to determine strength or weakness were missing. Findings of the relevance/credibility assessment are presented in Additional file 5.

The majority of cost-effectiveness analyses (28, 93%) utilised Markov models, and 20 of these (71%) used the Centre for Outcomes Research and Evaluation (CORE) Diabetes Model. Five studies used ‘author developed’ Markov models, three used the Sheffield Type 1 Diabetes Policy Model (patient-level Markov model), and another two were trial based. Nine of the cost-effectiveness analyses were reported from the societal perspective, and 22 were reported from the healthcare funder’s perspective in the base case. Real option analysis with cash flow simulations took the market perspective.

**Fig. 2:**
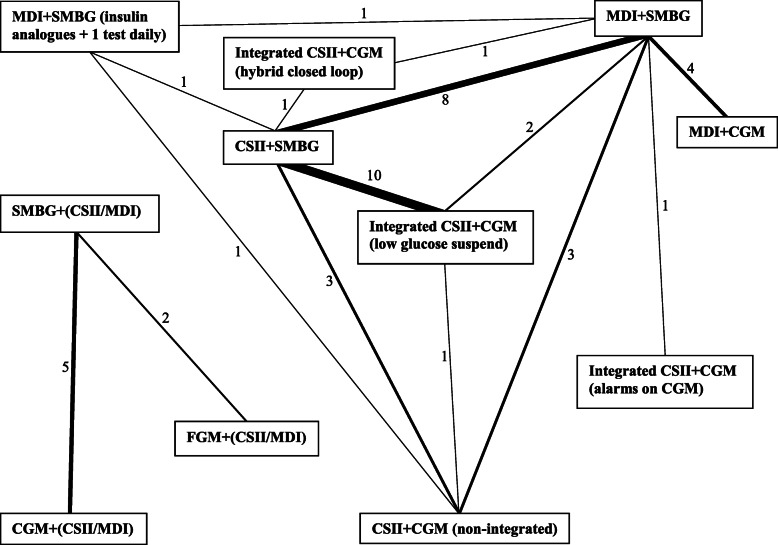
Network map of diabetes management technologies compared directly by economic evaluations. Each box contains a diabetes management technology and each line indicates direct comparison(s) by economic evaluation(s) retrieved by our systematic review. The width of each line and numbers indicate how many economic evaluations have compared technology types

A number of country settings were considered, including ten economic evaluations from the United Kingdom (UK, 29%), nine from Europe (26%), eight from the United States of America (USA, 23%), four from Canada (11%), two from Australia (6%), two from Spain (6%), and one from Colombia (3%). One publication from Canada presented a cost-effectiveness evaluation and separate budget impact analysis. Technologies with economic evaluation(s) included MDI, CSII, SMBG, CGM, flash glucose monitoring (FGM), as well as integrated CSII with CGM (utilising alarms, low glucose suspend, or a hybrid closed loop system). See Fig. 2 for the network of compared technologies.

After adjustment for inflation, patient time costs were reported as $1821 USD (95% confidence interval (CI) 829–2768) higher for CSII and CGM compared to MDI and SMBG over a 1-year period of intervention in the USA [26]. Cost-calculations of CGM compared with SMBG (using either CSII or MDI) indicated a cost saving of $1025–$1458 USD over a 1-year period [27]. Flash glucose monitoring was reported to provide $474 AUD ($333 USD) cost savings over 1 year compared to SMBG and heterogeneous insulin delivery methods in the UK [28], based on the ‘Novel Glucose-Sensing Technology and Hypoglycemia in Type 1 Diabetes: a Multicentre, Non-masked, Randomised Controlled Trial’ (IMPACT study) [29]. A budget impact analysis in the UK reported CGM to have ‘minimal budget impact’ compared to SMBG when insulin delivery modality was either CSII or MDI [30]. The real option analysis approach with cash flow simulation of a cohort in Finland reported that integrated CSII and CGM systems provide $1,000,792 AUD ($704,029 USD) cost savings in comparison to MDI with SMBG over a lifetime [25].

Of the 35 studies eligible for analysis, 26 received funding or material support from manufacturers of insulin pumps or glucose sensors. The majority of these (22, 85%) reported base case ICERs below willingness-to-pay thresholds, were deemed to have minimal impact on budget, or to reduce costs in cost-calculations. A component of non-commercial funding was reported for 12 of the studies from our review. Four of these reported a favourable economic evaluation for the intervention arms, of which three also received a component of commercial funding or material support from manufacturers of diabetes management technologies. All of the nine studies with base case results from the societal perspective reported advanced technology to be cost-effective although sensitivity analyses including only direct costs had minimal impact on conclusions. Overall, there were 24 (69%) studies that reported favourable economic evaluations for the intervention arms. ICERs per life year gained were infrequently reported, and results were similar to ICERs per QALY gained.

Freestanding interstitial-glucose monitoring appeared to be cost-effective in eight of 13 (62%) studies prior to inflation, although ICERs ranged widely. Two of four studies reported that CGM and MDI were cost-effective when compared to SMBG and MDI. When adjusted for inflation, ICERs for CGM and MDI ranged from $37,470 AUD ($26,361 USD) per QALY gained in the Canadian setting [31] through to $1,224,807 AUD ($861,686 USD) per QALY reported by Health Quality Ontario [32]. When insulin delivery comprised either CSII or MDI, four of five economic evaluations favoured CGM over SMBG, and ICERs ranged from $79,161 AUD ($55,692 USD) per QALY gained in the USA [16] through to $4,261,481 AUD ($2,997,832 USD) per QALY in Spain [33]. When all participants utilised CSII therapy, non-integrated CGM was cost-effective in one of three studies compared to SMBG, and ICERs ranged from $61,129 AUD ($43,003 USD) per QALY gained in the Swedish context [34] through to $1,350,689 AUD ($950,245 USD) per QALY in the UK [17]. FGM was cost-effective compared to SMBG with insulin delivery comprising CSII or MDI, and the adjusted ICER was $46,705 AUD ($32,857 USD) per QALY gained in Sweden [35].

When compared to CSII and SMBG, integrated systems of CSII and CGM with a suspend function for low glucose were reported as cost-effective for eight of ten (80%) relevant studies at the time of their publication. When adjusted for inflation and exchange rates, the ICERs for studies reporting that integrated systems were cost-effective ranged from $19,695 AUD ($14,266 USD) per QALY gained in Denmark for participants at risk of hypoglycaemia [36] through to $75,025 AUD ($52,778 USD) per QALY in Italy for participants with HbA1c levels > 8.0% (64 mmol/mol) [37]. Studies that concluded integrated systems were not cost-effective reported ICERs of $656,037 AUD ($461,540 USD) per QALY in Canada [32] and $1,477,075 AUD ($1,039,160 USD) per QALY in the UK [17]. Integrated CSII and CGM systems were reported as not cost-effective in the only comparison to non-integrated systems [17]. Use of a hybrid closed loop system was considered cost-effective when compared to CSII and SMBG in the Swedish context with an adjusted ICER of $25,327 AUD ($17,817 USD) per QALY gained [38].

CSII and SMBG were reported as cost-effective when compared to MDI and SMBG in five of nine (56%) studies. ICERs below willingness-to-pay thresholds, when adjusted for inflation and currency, ranged from $29,860 AUD ($21,008 USD) [39] per QALY gained to $81,082 AUD ($57,043 USD) per QALY in the USA [24]. Studies with ICERs above willingness-to-pay thresholds ranged from $91,356 AUD ($64,271 USD) [40] to $291,685 AUD ($205,208 USD) per QALY gained [41] both of which were set in the UK.

The comparisons of MDI and SMBG with either non-integrated or integrated CSII and CGM systems (low glucose suspend or CGM alarm features) reported that modern technologies were generally not cost-effective. Non-integrated systems were reported as not cost-effective when compared to MDI with SMBG, with an adjusted ICER of $383,717 AUD ($269,955 USD) per QALY gained in the USA [42]. Integrated systems with a low glucose suspend feature were also reported as not cost-effective, with adjusted ICERs of $249,465 AUD ($175,505 USD) [17] and $1,114,930 AUD ($784,384 USD) [32] per QALY gained in the UK and Canada, respectively. The single article that considered CGM alarm features reported it was cost-effective compared to MDI and SMBG, with an adjusted ICER of $43,694 AUD ($30,740 USD) per QALY gained in the Colombian setting [43]. Herman et al. reported on the comparison of modern intensive treatments (MDI with SMBG, CSII with non-integrated CGM, and CSII with SMBG) to ‘basic’ MDI and SMBG therapy limited to one capillary glucose test per day [24]. Compared to basic therapy, CSII with SMBG was cost-effective, with an ICER of $81,082 AUD ($57,043 USD) but non-integrated CSII and CGM was not cost-effective, with an ICER of $410,317 AUD ($288,669 USD) per QALY gained [24].

We developed a visual network of comparisons from the literature (Fig. 2) and found that technologies were not compared with the full spectrum of available alternatives in economic evaluations. The most commonly compared technologies were CSII and SMBG versus MDI and SMBG, or the comparison of integrated systems with low glucose suspend versus CSII with SMBG. There was no comparison of CGM and MDI with any system other than SMBG and MDI. Integrated systems with CGM alarms were not compared to systems with low glucose suspend, and only one study compared non-integrated CSII and CGM with an integrated system. Furthermore, hybrid closed loop systems have only been considered in one cost-effectiveness analysis compared to CSII with SMBG. Relatively less advanced technologies such as insulin dose calculators or smart-device applications have not to date been evaluated from an economic perspective.

### Modelled treatment effects of diabetes management technology

Up until 2009, all economic evaluations compared CSII to MDI and modelled an HbA1c reduction of 1.2% (13.1 mmol/mol) in favour of CSII based on the meta-analysis by Weissberg-Benchell et al. [44]. Sensitivity analyses among economic evaluations also modelled the impact of reducing the treatment effect of HbA1c to 0.51% (5.6 mmol/mol) as suggested by the meta-analysis of Pickup et al. [45]. Authors of these economic evaluations assumed no difference in rates of hypoglycaemia in the base case, but sensitivity analyses modelled the impact of a 50–75% reduction in hypoglycaemia. The article by Cummins et al modelled a 0.9% (9.8 mmol/mol) reduction in HbA1c values from CSII therapy, citing submitted but unpublished data by ‘R. Feltbower and the Database group, April 2007’ [40]. The most recent article comparing CSII to MDI modelled HbA1c reduction of 0.24% (2.6 mmol/mol), citing the ‘Relative effectiveness of insulin pump treatment over multiple daily injections and structured education during flexible intensive insulin treatment for type 1 diabetes: cluster randomised trial (REPOSE)’ study [46].

Comparisons of CGM to SMBG cited HbA1c reductions ranging from 0.23% (2.5 mmol/mol; from Garcia-Lorenzo et al.’s meta-analysis for their own economic evaluation) [33] to 0.6% (6.6 mmol/mol), citing the ‘Effect of continuous glucose monitoring on glycemic control in adults with type 1 diabetes using insulin injections: the DIAMOND randomized clinical trial’ (DIAMOND) study [47]. The treatment effect of CGM on severe hypoglycaemia ranged from no impact (author assumption) through to a 50% reduction in the rate of events [48]. The only cost-effectiveness analysis for FGM compared to SMBG assumed equivalent HbA1c effects and rates of severe hypoglycaemia between treatment groups [35]. However, the group using FGM had 4897.10 non severe hypoglycaemic events per 100 person-years compared to 6760.00 events per 100 person-years in the SMBG group [35].

Treatment effects for a hybrid closed loop system were based on a pivotal trial and before-and-after study without comparator arms [49, 50]. The hybrid closed loop system was modelled to reduce HbA1c by 0.5% (5.5 mmol/mol) [49] and was assumed to prevent all episodes of severe hypoglycaemia over a lifetime [38]. For economic evaluation, the comparator of CSII and SMBG was assumed to have no impact on HbA1c, and severe hypoglycaemia was modelled to occur at a rate of 25 events per 100 person-years requiring medical assistance and 65 events per 100 person-years requiring non-medical assistance [38].

Hypoglycaemia for integrated systems with low glucose suspend were usually modelled as no events compared to 2.2 events per 100 patient months for CSII and SMBG, based on the RCT by Ly et al. [51]. Reduction of HbA1c for integrated systems compared to CSII with SMBG was mostly modelled on results of the patient-level meta-analysis by Pickup et al. [52]. Utilised values from this meta-analysis ranged from the reported overall HbA1c reduction of 0.3% (3.3 mmol/mol) through to 0.9% (9.8 mmol/mol) if the baseline value was 10% (86 mmol/mol) [52].

The only economic evaluation that considered integrated systems with CGM alarms compared to MDI with SMBG utilised their own review article (in Spanish) as the source for their clinical assumptions [43]. Authors reported the largest treatment effect of interventions from any study, citing HbA1c reductions of 1.5% (16.4 mmol/mol) and severe hypoglycaemia rates reducing from 5.22 to 0.37 events per patient/year [43].

### Determinants of cost-effectiveness

The comparative effectiveness of various diabetes management technologies was largely based on either reduction of HbA1c values or reduced rates of hypoglycaemia. Rates of hypoglycaemia were utilised to variably estimate long-term costs incurred from lost productivity and the direct costs of ambulance and hospital admissions. However, HbA1c was the primary basis for predicting long-term complication rates and therefore contributed largely to estimates of cost. This was exemplified by sensitivity analyses, in which an increase in relative treatment effect on HbA1c from 0.6% (6.6 mmol/mol; base case) to 1.2% (13.1 mmol/mol) for non-integrated CSII and CGM systems reduced ICERs from $229,675 to $29,037 (2010 USD) per QALY gained [42]. Furthermore, the economic evaluation by Riemsma et al. concluded that CSII with or without CGM was not cost-effective, but reported small HbA1c reductions of 0.06% (0.7 mmol/mol) for integrated CSII and CGM (Vibe TM) systems through to an HbA1c increment of 0.05% (0.5 mmol/mol) for CSII with SMBG and 0.64% (7 mmol/mol) for MDI and SMBG [17]. Alteration of baseline HbA1c values also impacted ICER values when utilising the patient level meta-analysis by Pickup et al. due to increasing treatment effects reported for participants with worse baseline glycaemic control [52]. For example, the base case ICER of 156,082 (2016 Denmark krone [DKK]) reduced to 116,755 (2016 DKK) per QALY gained in the analysis by Roze et al. when the baseline HbA1c was increased from 8.1% (65 mmol/mol) to 9.0% (75 mmol/mol) in sensitivity analysis [36].

Apart from the impact of clinical effectiveness on costs, international health systems valued complications differently and authors variably determined utility weights. For example, the costing of ischaemic heart disease varied from $4,486 (2003 GBP) [53] to $35,271 (2007 USD) [16] and the cost of vision loss ranged from €358 (2014 EUR) [54] to $9,912 (2007 USD) [16]. Furthermore, studies comparing CSII to MDI reported differences in QALYs ranging from 0.47 to 1.06 [39, 55]. Non-integrated CSII and CGM had QALYs reported as 0.38 higher than MDI and SMBG [42]. Integrated systems with low glucose suspend had QALYs 0.04–2.99 higher than CSII with SMBG [51, 56]. The largest difference in QALYs was reported by Gomez et al., with integrated systems involving CGM alarms being 3.81 higher than MDI with SMBG [43]. Sensitivity analyses by Ly et al. reported ICERs of $40,803 (AUD 2013) per QALY which increased to $382,954 (AUD 2013) per QALY for a utility value of 0.0075, and decreased to $21,565 (AUD 2013) per QALY with a utility value of 0.1390 [51].

## Discussion

This review of 35 economic evaluations comprising 11 management strategies and 14 technology comparisons found variable results. While most studies favoured the cost-effectiveness of advanced diabetes management technologies, some comparisons had few studies, and ICERs ranged widely below and above willingness-to-pay thresholds.

Estimates of cost-effectiveness were sensitive to glycaemic indicators such as the treatment impact on HbA1c and rates of hypoglycaemia as well as baseline glycaemic control. Base case scenarios often modelled the effect of HbA1c reduction alone, applying the conservative assumption that hypoglycaemia rates were equivalent between groups. The majority of studies reporting that CSII or CGM technology was cost-effective modelled a treatment effect for HbA1c greater than 1.0% (10.9 mmol/mol) and 0.5% (5.5 mmol/mol), respectively. When the base-case modelled only one aspect of glycaemic control, sensitivity analyses that included both HbA1c reduction and prevention of hypoglycaemia often decreased ICERs below willingness-to-pay thresholds. Furthermore, our finding that CSII technology was cost-effective across multiple settings is in keeping with prior reviews [13, 40].

Data sources for the glycaemic impact of technologies had significant potential to impact results of cost-effectiveness analyses. Economic evaluations of CSII technology compared to MDI modelled HbA1c reductions ranging from 0.51% (5.6 mmol/mol) to 1.2% (13.1 mmol/mol), citing meta-analyses by Pickup et al. and Weissberg-Benchell et al. respectively [44, 45]. These were limited by inclusion of paediatric participants in some studies from both meta-analyses, and the analysis by Weissberg-Benchell et al. included some observational studies [44]. Another two studies reported modelled HbA1c coefficients that could not be interpreted but were based on the REPOSE study which reported an HbA1c adjusted mean difference of 0.24% (2.6 mmol/mol) favouring CSII therapy [46]. This study excluded participants with a preference for CSII therapy which may have impacted the effect size and generalisability of results. Herman et al. utilised CSII and MDI treatment effects based on the intensive and conventional treatment arms of the landmark diabetes control and complications trial (DCCT) published in 1995 which may not reflect contemporary management strategies or HbA1c treatment effects [24, 57]. Economic evaluations that compared CGM to SMBG modelled treatment effects for HbA1c as a reduction of 0.23% (2.5 mmol/mol) to 0.6% (6.6 mmol/mol) [30, 33]. Three studies that reported CGM was not cost-effective modelled a treatment effect for HbA1c less than 0.5% (5.5 mmol/mol) from the authors’ own respective meta-analyses [14, 17, 33]. The only other study that reported CGM (Health Quality Ontario 2018) was not cost-effective modelled an HbA1c treatment effect > 0.5% (5.5 mmol/mol) but did not assume lifelong effectiveness or risk reduction for diabetes related complications in the base-case [32].

Severe hypoglycaemia also exerted a large impact on cost-effectiveness, but the chosen data sources may not have been transferrable to long-term modelling. The approaches to hypoglycaemia included the assumption of equivalent rates between management strategies or utilising meta-analysis and RCT estimates ranging from 13.1 (citing Bergenstal et al. [58]) to 84.7 (citing Bode et al. [59]) rate reduction for advanced technologies. The Australian study by Ly et al. that included paediatric participants was often cited, concluding that no events occurred while using integrated systems with low glucose suspend, and 2.2 events per 100 patient months occurred while using CSII and SMBG [60]. The only cost-effectiveness analysis of a hybrid closed loop system cited two studies that found no episodes of hypoglycaemia over 3-month study periods [49, 50]. The resultant assumption of no severe hypoglycaemia over a modelled lifetime horizon among users of hybrid closed loop systems or low glucose suspend systems was a key driver of cost-effectiveness in these studies. While the approach to sensitivity analyses for hypoglycaemia was also variable among the studies, increasing baseline rates of hypoglycaemia or treatment effect for hypoglycaemia prevention reduced ICERs considerably when compared to the base-case. However, no clinical trial comparing technology in diabetes management has so far been adequately powered to make strong conclusions about treatment effects on severe hypoglycaemia. Furthermore, the short duration of cited RCTs for severe and non-severe hypoglycaemia may not be generalisable to decades-long modelled time horizons that may otherwise overestimate the effectiveness of advanced technologies.

Apart from the clinical impact of management strategies, the costing of diabetes management technologies and the complications of diabetes varied across international settings. The generalisability and transferability of results from economic evaluations may therefore be limited primarily to the healthcare systems in which they were performed. Despite authors citing valid sources for the utility and disutility weights of diabetes and numerous complications, these values also varied across studies with no consensus in the literature. This was of particular relevance due to the crucial role of QALYs in deriving ICERs. For example, the utility for fear of hypoglycaemia was a key driver of cost-effectiveness for integrated systems with low glucose suspend in the base-case scenarios where glycaemic effects were assumed to be equivalent or only marginally different to the control arm. Similarly for flash glucose monitoring, the utility increment associated with a 25.8% reduction in non-severe hypoglycaemia was a key driver of cost-effectiveness while modelling equivalent HbA1c and severe hypoglycaemia event rates [35]. However, while differences in costing and utility weights limit pooling of cost-effectiveness estimates across different healthcare systems, the factors that contributed to cost-effectiveness for individual studies provide insight to guide decisions internationally.

Our study is the largest review of economic evaluations to date, with the broadest consideration of technologies in the management of type 1 diabetes. While pooling of cost-effectiveness data was not possible, we were able to summarise the existing landscape of economic evidence and outline common factors present among technologies considered cost-effective in various settings. Furthermore, we followed a systematic approach outlined in our published protocol, and reported our review in line with the PRISMA statement guidelines [19]. Limitations included restricting our search to English language and not systematically including ‘grey literature’. Probabilistic sensitivity analyses and confidence intervals around economic results would have strengthened the majority of studies in order to help clarify the relative contribution of key variables to overall economic uncertainty. Furthermore, patient preferences and the possibility of reverting to basic management strategies or trying other forms of technology were not completely captured by any of the included modelling studies. In the absence of a gold standard approach, appraisal of the quality of reviewed studies utilised the tool outlined by ISPOR due to the large number of modelling studies [61]. This process includes subjective assessments and did not generate an overall quality score, but independent duplication of the appraisal process with two reviewers reaching consensus decisions addressed this issue in our analysis. Appraisal systems that generate scores also suffer from limitations regarding weighting for some criteria, masking low scores in some domains, binary responses to multifaceted issues, and the absence of ‘fatal flaw’ criteria. This approach to appraisal differed from our protocol due to the significantly larger volume and variety of economic evaluations than had been noted in prior reviews. Because only one study considered the perspective of a developing country, we were also unable to perform subgroup analyses based on gross domestic product to further clarify generalisability of international results apart from inflation/deflation.

## Conclusions

Most studies in our network reported that newer diabetes management technologies were cost-effective although ICERs varied widely. Insulin pumps or glucose sensors appeared cost-effective, especially in populations with the most to gain from such interventions such as those with higher HbA1c levels and rates of hypoglycaemia. Results for combined insulin pump and glucose-sensing technology was less clear, although hybrid closed loop therapy and systems with low glucose suspend appeared cost-effective in comparison to CSII and SMBG. An important limitation is that pump therapy is not universally funded by many countries which highlights the need for economic evaluations that compare integrated systems to the more ubiquitous standard of care comprising MDI and SMBG.

Notwithstanding the limitations of the evidence base, our systematic review and narrative synthesis provides the most comprehensive and contemporary evidence to guide economic comparison between diabetes management technologies. While it was not feasible to quantitatively compare data across studies, we believe that uniformity of reporting costs and utilities would greatly assist in comparing economic evaluations within healthcare systems. Furthermore, the rapid pace of technology development means that studies struggle to remain current. We therefore advocate for economic evaluations of all clinically relevant combinations of technology in different healthcare systems as well as the adoption of living systematic reviews to facilitate rapid incorporation of evidence into clinical practice guidelines. A current focus should be on economic evaluations of closed loop systems and comparison with the most ubiquitous treatment strategy which, for many countries remains multiple daily injections and capillary blood glucose testing.

## Supplementary information

**Additional file 1.** PRISMA 2009 Checklist. Description: Completed PRISMA 2009 checklist.

**Additional file 2.** Supplementary appendix. Description: Reference list of included records and study identifiers from the systematic review.

**Additional file 3.** Table 1. Treatments, treatment effects, costs, and quality adjusted life years. Description: Table outlining the details of treatment comparisons, modelled treatment effects, cited costs, and reported quality adjusted life years as appropriate.

**Additional file 4.** Table 2. Study summaries. Description: Table outlining summaries of each study in the systematic review.

**Additional file 5.** Table 3. Assessment of study relevance and credibility. Description: Table outlining the assessment of relevance and credibility for each study in the systematic review.

## Data Availability

The datasets used and/or analysed during the current study are available from the corresponding author on reasonable request.

## Abbreviations

AMCP: Academy of Managed Care Pharmacy
AUD: Australian dollars
BMI: Body mass index
CAN: Canadian dollars
CGM: Continuous glucose monitor
CI: Confidence interval
COI: Conflict of interest
COP: Colombian peso
CORE: Centre for Outcomes Research and Evaluation
CSII: Continuous subcutaneous insulin infusion
DAFNE: Dose adjustment for normal eating
DCCT: Diabetes control and complications trial
DKA: Diabetic ketoacidosis
DKK: Denmark krone
EDIC: Epidemiology of diabetes interventions and complications
EEACT: Economic evaluation alongside clinical trials
EUR: Euros
FGM: Flash glucose monitor
GBP: British pound sterling
ICER: Incremental cost effectiveness ratio
ID: Identifier
IMS: Information Medical Statistics
ISPOR: International Society for Pharmacoeconomics and Outcomes Research
JDRF: Juvenile Diabetes Research Foundation
MDI: Multiple daily injections
NICE: National Institute for Health and Clinical Excellence
NPC: National Pharmaceutical Council
PRISMA: Preferred reporting items for systematic reviews and meta-analyses
QALE: Quality adjusted life expectancy
QALY: Quality adjusted life year
RCTs: Randomised controlled trials
SD: Standard deviation
SEK: Swedish krona
SMBG: Self-monitoring of capillary blood glucose
UK: United Kingdom
USA: United States of America
USD: United States dollars
