# Supplementary material for: Cost-effectiveness of health technologies in adults with type 1 diabetes: a systematic review and narrative synthesis
Source: Syst Rev. 2020 Aug 3;9:171. doi: 10.1186/s13643-020-01373-y (PMC7401226; doi:10.1186/s13643-020-01373-y)
Supplement: Supplementary file 2 — Additional file 2. Supplementary appendix. Description: Reference list of included records and study identifiers from the systematic review. [file 13643_2020_1373_MOESM2_ESM.docx]

**Supplementary appendix**

**Reference list of included records and study identifiers:**

Scuffham 2003

- Scuffham P, Carr L. The cost-effectiveness of continuous subcutaneous insulin infusion compared with multiple daily injections for the management of diabetes. Diabet Med 2003; 20: 586–593.

Roze 2005

- Roze S, Valentine WJ, Zakrzewska KA, Palmer AJ. Health-economic comparison of continuous subcutaneous insulin infusion with multiple daily injection for the treatment of type 1 diabetes in the UK. Diabet Med 2005; 22: 1239–1245.

Cohen 2007

- Cohen N, Minshall ME, Sharon-Nash L, Zakrzewska K, Valentine WJ, Palmer AJ. Continuous subcutaneous insulin infusion versus multiple daily injections of insulin: economic comparison in adult and adolescent type 1 diabetes mellitus in Australia. Pharmacoeconomics 2007; 25: 881–897.

St Charles 2009a

- St Charles M, Lynch P, Graham C, Minshall ME. A cost-effectiveness analysis of continuous subcutaneous insulin injection versus multiple daily injections in type 1 diabetes patients: a third-party US payer perspective. Value Health 2009; 12: 674–686.

St Charles 2009b

- St Charles ME, Sadri H, Minshall ME, Tunis SL. Health economic comparison between continuous subcutaneous insulin infusion and multiple daily injections of insulin for the treatment of adult type 1 diabetes in Canada. Clin Ther 2009; 31: 657–667.

Cummins 2010

- Cummins E, Royle P, Snaith A, et al. Clinical effectiveness and cost-effectiveness of continuous subcutaneous insulin infusion for diabetes: systematic review and economic evaluation. Health Technol Assess 2010; 14: iii–iv, xi–xvi, 1–181.

Huang 2010

- Huang ES, O’Grady M, Basu A, et al. The cost-effectiveness of continuous glucose monitoring in type 1 diabetes. Diabetes Care 2010; 33: 1269–1274.

McQueen 2011

- McQueen RB, Ellis SL, Campbell JD, Nair KV, Sullivan PW. Cost-effectiveness of continuous glucose monitoring and intensive insulin therapy for type 1 diabetes. Cost Eff Resour Alloc 2011; 9:13. doi: 10.1186/1478-7547-9-13.

Kamble 2012

- Kamble S, Schulman KA, Reed SD. Cost-effectiveness of sensor-augmented pump therapy in adults with type 1 diabetes in the United States. Value Health 2012; 15: 632–638.

Kamble 2013

- Kamble S, Weinfurt KP, Schulman KA, Reed SD. Patient time costs associated with sensor-augmented insulin pump therapy for type 1 diabetes: results from the STAR 3 randomized trial. Med Decis Making 2013; 33: 215–224.

Ly 2014

- Ly TT, Brnabic AJ, Eggleston A, et al. A cost-effectiveness analysis of sensor-augmented insulin pump therapy and automated insulin suspension versus standard pump therapy for hypoglycemic unaware patients with type 1 diabetes. Value Health 2014; 17: 561–569.

NICE 2015

- National Clinical Guideline Centre (UK). Type 1 Diabetes in Adults: Diagnosis and Management. London: National Institute for Health and Care Excellence (UK); (NICE Guideline, No. 17.), 2015. <https://www.ncbi.nlm.nih.gov/books/NBK343354/> (accessed Feb 15, 2019).

Roze 2015

- Roze S, Saunders R, Brandt AS, de Portu S, Papo NL, Jendle J. Health-economic analysis of real-time continuous glucose monitoring in people with type 1 diabetes. Diabet Med 2015; 32: 618–626.

Bronstone 2016

- Bronstone A, Graham C. The potential cost implications of averting severe hypoglycemic events requiring hospitalization in high-risk adults with type 1 diabetes using real-time continuous glucose monitoring. J Diabetes Sci Technol 2016; 10: 905–913.

Gomez 2016

- Gomez AM, Alfonso-Cristancho R, Orozco JJ, et al. Clinical and economic benefits of integrated pump/CGM technology therapy in patients with type 1 diabetes in Colombia. Endocrinol Nutr 2016; 63: 466–474.

Haahtela 2016

- Haahtela TJ. Real option approach for comparing lifetime costs of alternative diabetes type 1 treatment methods. Fuzzy Economic Review 2016; 21: 71–91.

Riemsma 2016

- Riemsma R, Corro Ramos I, Birnie R, et al. Integrated sensor-augmented pump therapy systems [the MiniMed® Paradigm™ Veo system and the Vibe™ and G4® PLATINUM CGM (continuous glucose monitoring) system] for managing blood glucose levels in type 1 diabetes: a systematic review and economic evaluation. Health Technol Assess 2016; 20: v–xxxi, 1–251.

Roze 2016a

- Roze S, Smith-Palmer J, Valentine WJ, et al. Long-term health economic benefits of sensor-augmented pump therapy vs continuous subcutaneous insulin infusion alone in type 1 diabetes: A U.K. perspective. J Med Econ 2016; 19: 236–242.

Roze 2016b

- Roze S, Smith-Palmer J, Valentine W, et al. Cost-effectiveness of sensor-augmented pump therapy with low glucose suspend versus standard insulin pump therapy in two different patient populations with type 1 diabetes in France. Diabetes Technol Ther 2016; 18: 75–84.

Chaugule 2017a

- Chaugule S, Graham C. Cost-effectiveness of G5 mobile continuous glucose monitoring device compared to self-monitoring of blood glucose alone for people with type 1 diabetes from the Canadian societal perspective. J Med Econ 2017; 20: 1128–1135.

Chaugule 2017b

- Chaugule S, Oliver N, Klinkenbijl B, Graham C. An economic evaluation of continuous glucose monitoring for people with type 1 diabetes and impaired awareness of hypoglycaemia within North West London clinical commissioning groups in England. Eur Endocrinol 2017; 13: 81–85.

Heller 2017

- Heller S, White D, Lee E, et al. A cluster randomised trial, cost-effectiveness analysis and psychosocial evaluation of insulin pump therapy compared with multiple injections during flexible intensive insulin therapy for type 1 diabetes: the REPOSE trial. Health Technol Assess 2017; 21: 1–278.

Jendle 2017

- Jendle J, Smith-Palmer J, Delbaere A, et al. Cost-effectiveness analysis of sensor-augmented insulin pump therapy with automated insulin suspension versus standard insulin pump therapy in patients with type 1 diabetes in Sweden. Diabetes Ther 2017; 8: 1015–1030.

Roze 2017

- Roze S, de Portu S, Smith-Palmer J, Delbaere A, Valentine W, Ridderstråle M. Cost-effectiveness of sensor-augmented pump therapy versus standard insulin pump therapy in patients with type 1 diabetes in Denmark. Diabetes Res Clin Pract 2017; 128: 6–14.

Bilir 2018

- Bilir SP, Hellmund R, Wehler B, Li H, Munakata J, Lamotte M. Cost-effectiveness analysis of a flash glucose monitoring system for patients with type 1 diabetes receiving intensive insulin treatment in Sweden. Eur Endocrinol 2018; 14: 73–79.

Conget 2018

- Conget I, Martín-Vaquero P, Roze S, et al. Cost-effectiveness analysis of sensor-augmented pump therapy with low glucose-suspend in patients with type 1 diabetes mellitus and high risk of hypoglycemia in Spain. Endocrinol Diabetes Nutr 2018; 65: 380–386.

García-Lorenzo 2018

- García-Lorenzo B, Rivero-Santana A, Vallejo-Torres L, et al. Cost-effectiveness analysis of real-time continuous monitoring glucose compared to self-monitoring of blood glucose for diabetes mellitus in Spain. J Eval Clin Pract 2018; 24: 772–781.

Health Quality Ontario 2018

- Health Quality Ontario. Continuous monitoring of glucose for type 1 diabetes: a health technology assessment. Ont Health Technol Assess Ser 2018; 18: 1–160.

Hellmund 2018

- Hellmund R, Weitgasser R, Blissett D. Cost calculation for a flash glucose monitoring system for UK adults with type 1 diabetes mellitus receiving intensive insulin treatment. Diabetes Res Clin Pract 2018; 138: 193–200.

Herman 2018

- Herman WH, Braffett BH, Kuo S, et al. The 30-year cost-effectiveness of alternative strategies to achieve excellent glycemic control in type 1 diabetes: an economic simulation informed by the results of the diabetes control and complications trial/epidemiology of diabetes interventions and complications (DCCT/EDIC). J Diabetes Complications 2018; 32: 934–939.

Nicolucci 2018

- Nicolucci A, Rossi MC, D’Ostilio D, Delbaere A, de Portu S, Roze S. Cost-effectiveness of sensor-augmented pump therapy in two different patient populations with type 1 diabetes in Italy. Nutr Metab Cardiovasc Dis 2018; 28: 707–715.

Pollard 2018

- Pollard DJ, Brennan A, Dixon S, et al. Cost-effectiveness of insulin pumps compared with multiple daily injections both provided with structured education for adults with type 1 diabetes: a health economic analysis of the relative effectiveness of pumps over structured education (REPOSE) randomised controlled trial. BMJ Open 2018; 8: e016766. doi: 10.1136/bmjopen-2017-016766.

Wan 2018

- Wan W, Skandari MR, Minc A, et al. Cost-effectiveness of continuous glucose monitoring for adults with type 1 diabetes compared With Self-Monitoring of Blood Glucose: The DIAMOND randomized trial. Diabetes Care 2018; 41: 1227–1234.

Jendle 2019

- Jendle J, Pöhlmann J, de Portu S, Smith-Palmer J, Roze S. Cost-effectiveness analysis of the MiniMed 670G hybrid closed-loop system versus continuous subcutaneous insulin infusion for treatment of type 1 diabetes. Diabetes Technol Ther 2019; 31: 110–118.

Roze 2019

- Roze S, Smith-Palmer J, de Portu S, Delbaere A, de Brouwer B, de Valk HW. Cost-effectiveness of sensor-augmented insulin pump therapy vs continuous subcutaneous insulin infusion in patients with type 1 diabetes in the Netherlands. Clinicoecon Outcomes Res 2019; 11: 73–82
