# Supplementary material for: Cost-effectiveness of health technologies in adults with type 1 diabetes: a systematic review and narrative synthesis
Source: Syst Rev. 2020 Aug 3;9:171. doi: 10.1186/s13643-020-01373-y (PMC7401226; doi:10.1186/s13643-020-01373-y)
Supplement: Supplementary file 3 — Additional file 3. Table 1. Treatments, treatment effects, costs, and quality adjusted life years. Description: Table outlining the details of treatment comparisons, modelled treatment effects, cited costs, and reported quality adjusted life years as appropriate. [file 13643_2020_1373_MOESM3_ESM.docx]

| **Table 1. Treatments, treatment effects, costs, and quality adjusted life years** | | | | | | | | | |
| --- | --- | --- | --- | --- | --- | --- | --- | --- | --- |
| **Study ID** | **Comparison / Treatment effects** | **Currency** | **Time horizon** | **Mean (SD) intervention cost** | **Mean (SD) control cost** | **Mean (SD) cost difference** | **Mean (SD) intervention QALY** | **Mean (SD) control QALY** | **Mean (SD) QALY difference** |
| Scuffham 2003 | CSII+SMBG vs MDI+SMBG  Base case treatment effects:  Risk reductions for CSII+SMBG: Mean (SD) rate of hypoglycaemic events per person per year 0.432 (0.177), admissions for hypoglycaemia 0.050 (0.020), diabetic ketoacidosis (DKA) 0.025 (0.010), insulin use 0.142 (0.057), additional CSII utility for CSII 0.053 (0.018), and monthly disutility with hypoglycaemia and DKA 0.067 (0.023). | GBP | 8 years | 9,514 (1,337) | 4,052 (1,792) | 5,462 (897) | 7.32 (0.39) | 6.85 (0.48) | 0.48 (0.20) |
| Roze 2005 | CSII+SMBG vs MDI+SMBG  Base case treatment effects:  Mean HbA1c reduction of 1.2% (13.1mmol/mol) and body mass index (BMI) increase of 1.03kg/m^2^ for CSII+SMBG. Hypoglycaemia and DKA rates were assumed equivalent between treatment groups. | GBP | 60 years | 80,511 (1,257) | 61,104 (1,249) | 19,407 (1,727) | Quality adjusted life expectancy (QALE) 12.03 (0.15) | QALE 11.27 (0.14) | QALE 0.76 (0.19) |
| Cohen 2007 | CSII+SMBG vs MDI+SMBG  Base case treatment effects:  Mean (SD) HbA1c reduction of 1.2% (0.2) [13.1mmol/mol (2.2)] and BMI increase of 1.03kg/m^2^ for CSII+SMBG. Hypoglycaemia and DKA rates were assumed equivalent between treatment groups. | AUD | 60 years | 123,402 (2,113) | 88,760 (2,055) | 34,642 | 7.950 (0.127) | 7.483 (0.090) | 0.467 |
| St Charles 2009a | CSII+SMBG vs MDI+SMBG  Base case treatment effects:  Mean (SD) HbA1c reduction of 1.2% (0.2) [13.1mmol/mol (2.2)] and BMI increase of 1.03kg/m^2^ for CSII+SMBG. CSII+SMBG was assumed to reduce hypoglycaemia rates by 50%. | USD | 60 years | 204,192 (2,950) | 186,170 (3,159) | 18,023 | 12.848 (0.197) | 11.788 (0.107) | 1.061 |
| St Charles 2009b | CSII+SMBG vs MDI+SMBG  Base case treatment effects:  Mean (SD) HbA1c reduction of 1.2% (0.2) [13.1mmol/mol (2.2)] and BMI increase of 1.03kg/m^2^ for CSII+SMBG. Hypoglycaemia rates were assumed equivalent between treatment groups. | Canadian dollars (CAN) | 60 years | 162,807 (3,544) | 147,216 (3,462) | 15,591 | 9.374 (0.076) | 10.029 (0.133) | 0.655 |
| Cummins 2010 | CSII+SMBG vs MDI+SMBG  Base case treatment effects:  CSII+SMBG modelled HbA1c reduction of 0.9% (9.8mmol/mol) [from 8.8% (73mmol/mol) to 7.9% (63mmol/mol)] and 50% reduction to the hypoglycaemia rate. | GBP | 50 years | General population with type 1 diabetes: 59,592  High risk for severe hypoglycaemia: 59,759 | General population with type 1 diabetes: 36,915  High risk for severe hypoglycaemia: 37,372 | General population with type 1 diabetes: 22,677  High risk for severe hypoglycaemia: 22,387 | General population with type 1 diabetes: 9.571  High risk for severe hypoglycaemia: 9.504 | General population with type 1 diabetes: 8.97  High risk for severe hypoglycaemia: 8.892 | General population with type 1 diabetes: 0.601  High risk for severe hypoglycaemia: 0.612 |
| Huang 2010 | CGM+(CSII/MDI) vs SMBG+(CSII/MDI)  Base case treatment effects:  CGM+(CSII/MDI):  Mean (SD) HbA1c: 7.1% (0.4) [54mmol/mol (4.4)]  Mean (SD) utility 0.8608 (0.0017)  SMBG+(CSII/MDI):  Mean (SD) HbA1c: 7.6% (0.4) [60mmol/mol (4.4)]  Mean (SD) utility 0.8838 (0.0005) | USD | Lifetime | 659,837  Results limited to the adult cohort (>25 years) with HbA1c >7.0% (53mmol/mol). | 601,070 | 58,767 | 14.35 | 13.75 | 0.60 |
| McQueen 2011 | CGM+(CSII/MDI) vs SMBG+(CSII/MDI)  Base case treatment effects:  CGM+(CSII/MDI) lead to HbA1c relative reduction of 0.5% (5.5mmol/mol).  Hypoglycaemia was not modelled. | USD | 33 years (cohort mean age of 40 years) | 494,135 (95% confidence interval [CI]: 420,381–571,631) | 470,583 (95% CI: 397,782 – 550,598) | 23,552 | 10.812 (95% CI: 9.894–11.887) | 10.289 (95% CI: 9.615–10.957) | 0.523 |
| Kamble 2012 | CSII+CGM vs MDI+SMBG  Base case treatment effects:  Mean (SD) HbA1c reductions:  CSII+CGM: -1.0 (0.7)  [-10.9mmol/mol (7.7)]  MDI+SMBG: -0.4 (0.8)  [4.4mmol/mol (8.7)]  Also assumed 65% use of 3-day sensors. | USD | 60 years | 253,493 (3,730) | 167,170 (3,058) | 86,324 (4,703) | 10.794 (0.108) | 10.418 (0.107) | 0.376 (0.143) |
| Kamble 2013 | CSII+CGM vs MDI+SMBG  Weeks 1–7:  1.9 hours (1.2–2.6) more per week were spent by those using CSII+CGM on diabetes related care.  Weeks 8–52:  1 hour (0.4–1.7) more per week were spent by those using CSII+CGM on diabetes related care. | USD | Trial period (52 weeks) | Wages only  4,600 (3,533)  Cumulative patient time:  Entire cohort:  222.6 (170.9)  Adult cohort:  221.5 (168.2) | Wages only  3,523 (3,318)  Cumulative patient time:  Entire cohort:  170.4 (160.5)  Adult cohort:  166.1 (159.5) | Wages only  1,077 (95% CI: 491 – 1,638) | NA | NA | NA |
| Ly 2014 | Integrated (low glucose suspend) vs CSII+SMBG  Base case treatment effects:  Incident rate difference for severe hypoglycaemia was 1.85 (0.17–3.53), in favour of integrated systems. | AUD | 6 months (trial period) | 4,431.84 | 2,929.32 | 1,502.52 | 0.03665 | - 0.00017 | 0.03682 |
| NICE 2015 | MDI+CGM vs MDI+SMBG (SMBG frequency: 2, 4, 6, 8, and 10 times daily).  Base case treatment effects:  MDI+CGM lowered HbA1c 0.30% more than MDI+SMBG at each frequency of blood sugar level (BSL) testing.  Hypoglycaemia event rates were assumed to be equivalent between groups. | GBP | Lifetime (80 years) | Mean (SD) discounted cost per patient:  Deterministic:  93,980 (24,132)  Probabilistic:  98,992 | Mean (SD) discounted cost per patient:  Deterministic:  SMBG 2: 41,805 (22,118)  SMBG 4: 41,989 (21,302)  SMBG 6: 43,685 (20,691)  SMBG 8: 46,288 (20,511)  SMBG 10: 49,146 (20,182)  Probabilistic:  44,075  41,856  42,692  44,517  47,062 | Cost difference:  Deterministic:  52,175  51,991  50,295  47,692  44,834  Probabilistic:  54,917  57,136  56,300  54,475  51,930 | Mean (SD) discounted QALY per patient:  Deterministic:  11.615 (3.539)  Probabilistic:  12.996 | Mean (SD) discounted QALY per patient:  Deterministic:  SMBG 2: 10.808 (3.654)  SMBG 4: 11.397 (3.575)  SMBG 6:  11.715 (3.521)  SMBG 8:  11.908 (3.481)  SMBG 10:  12.03 (3.539)  Probabilistic:  12.1  12.752  13.103  13.344  13.492 | QALY difference:  Deterministic:  0.807  0.218  -0.100  -0.293  -0.415  Probabilistic:  0.896  0.244  -0.107  -0.348  -0.496 |
| Roze 2015 | CSII+CGM vs CSII+SMBG  Base case treatment effects:  HbA1c was 0.3% (3.3mmol/mol) lower, and exposure to hypoglycaemia reduced by 23% among participants using CSII+CGM.  CSII+CGM: Mean of 4.4 BSL tests per day.  CSII+SMBG: Mean of 7.1 BSL tests per day. | Swedish Krona (SEK) | Lifetime | 2,872,525 (64,226) | 2,592,563 (63,127) | 279,962 | 13.05 (0.12) | 12.29 (0.12) | 0.76 |
| Bronstone 2016 | CGM+(CSII/MDI) vs SMBG+(CSII/MDI)  Base case treatment effects:  CGM+(CSII/MDI) reduced severe hypoglycaemia rates by 32%. Assumed HbA1c levels were equivalent between groups. | USD | 1 year | Episodes:  37,944  Hospitalisations:  5,502  Cost:  115,542,000 | Episodes:  55,800  Hospitalisations:  8,091  Cost: 169,911,000 | Cost reduction for hospital visits:  54,369,000  Cost saving (cost reduction from fewer hospital visits minus cost of CGM):  8,799,000 to 12,519,000.  946–1,346 per patient. | N/A | N/A | N/A |
| Gomez 2016 | Integrated (with alarms) vs MDI+SMBG  Base case treatment effects:  Integrated system reduced HbA1c by 1.5% (16.4mmol/mol) and severe hypoglycaemia event rate from 5.22 to 0.37 per year. | Colombian pesos (COP)  USD | 55 years | Total: 317,984,776  Direct: 298,701,051  Total: 170,100  Direct: 159,800 | Total: 151,125,045  Direct: 127,879,569  Total: 80,800  Direct: 68,400 | Total: 166,859,782  Direct: 170,821,482  Total: 89,300  Direct: 91,400 | 19.63 (0.35) | 15.83 (0.25) | 3.81 |
| Haahtela 2016 | Integrated vs MDI+SMBG  Base case treatment effects:  Unclear modelled differences between technologies. | EUR (Finland) | Lifetime | 300,000  (total discounted costs without quality of life costs) | 900,000  (total discounted costs without quality of life costs) | 600,000 | N/A | N/A | N/A |
| Riemsma 2016 | MiniMed Paradign Veo (integrated with low glucose suspend)  vs  1. CSII+CGM  2. CSII+SMBG  3. MDI+CGM  4. MDI+SMBG  Vibe+G4 platinum CGM  vs  1. CSII+CGM  2. CSII+SMBG  3. MDI+CGM  4. MDI+SMBG  Base case treatment effects:  Mean (SD) HbA1c reduction by 0.02 (0.04) [0.2mmol/mol (0.4)] for MiniMed,  0.06 (0.05) [0.7mmol/mol (0.5)] for Vibe,  0.06 (0.05) [0.7mmol/mol (0.5)] for CSII+CGM (assumed).  HbA1c increased by  0.05 (0.12) [0.5mmol/mol (1.3)] for CSII+SMBG  0.64 (0.19) [7mmol/mol (2.1)] for MDI+SMBG.  Rate ratio per 100 patient years for severe hypos were 1.9584 for Veo, 16.32 for Vibe, 5.0215 for CSII+SMBG, 19.584 for MDI+SMBG, and 16.32 for CSII+CGM.  BSL testing was assumed to occur four times per day for all participants. Insulin doses were also assumed to be equivalent between groups. | GBP | 80 years | MiniMed: 138,357  Vibe: 147,150 | CSII+CGM: 146,476  CSII+SMBG: 90,436  MDI+CGM: N/A  MDI+SMBG: 61,050  CSII+CGM: 146,476  CSII+SMBG: 90,436  MDI+CGM: N/A  MDI+SMBG: 61,050 | Incremental costs compared to MiniMed Paradigm Veo:  CSII+CGM: 8,119  CSII+SMBG: 47,921  MDI+CGM: N/A  MDI+SMBG: 77,307  Incremental costs compared to Vibe:  CSII+CGM: 674  CSII+SMBG: 56,713  MDI+CGM: N/A  MDI+SMBG: 86,100 | MiniMed: 12.0412  Vibe: 12,0604 | CSII+CGM: 12.0604  CSII+SMBG: 11.9756  MDI+CGM: N/A  MDI+SMBG: 11.4146  CSII+CGM: 12.0604  CSII+SMBG: 11.9756  MDI+CGM: N/A  MDI+SMBG: 11.4146 | Incremental QALYs compared to MiniMed Paradigm Veo:  CSII+CGM: -0.0192  CSII+SMBG: 0.0656  MDI+CGM: N/A  MDI+SMBG: 0.6266  Incremental QALYs compared to Vibe:  CSII+CGM: 0  CSII+SMBG: 0.0849  MDI+CGM: N/A  MDI+SMBG: 0.6458 |
| Roze 2016a | Integrated (low glucose suspend) vs CSII+SMBG  Base case treatment effects:  Integrated: Mean HbA1c reduction (-1.49% [16.3mmol/mol]), 0 severe hypoglycaemia events per 100 patient months, and utility benefit of 0.0552 due to less fear of hypoglycaemia.  CSII+SMBG: Mean HbA1c reduction (-0.62% [6.8mmol/mol]), and 2.2 severe hypoglycaemia events 100 patient months. | GBP | Lifetime | 125,559 | 88,991 | 36,568 | 17.88 | 14.89 | 2.99 |
| Roze 2016b | Integrated (low glucose suspend) vs CSII+SMBG  Base case treatment effects:  Group with elevated blood glucose:  Integrated: Mean HbA1c reduction of 0.88% (9.6mmol/mol) and utilised mean 4.35 BSL test strips per day.  CSII+SMBG: Mean HbA1c reduction of 0.48% (5.2mmol/mol) and utilised mean 7.11 BSL test strips per day.  Equivalent rates of severe hypoglycaemia between groups (2.6 events per 100 patient years).  Group at risk of hypoglycaemia:  Integrated: 0 events of severe hypoglycaemia per 100 patient months.  CSII+SMBG: 2.2 events of severe hypoglycaemia per 100 patient months.  Strip use was equivalent between groups. | EUR | Lifetime | Group with elevated blood glucose:  84,972  Group at risk of hypoglycaemia:  88,680 | Group with elevated blood glucose:  49,171  Group at risk of hypoglycaemia:  57,097 | Group with elevated blood glucose:  35,801  Group at risk of hypoglycaemia:  31,583 | Group with elevated blood glucose:  10.55  Group at risk of hypoglycaemia:  11.67 | Group with elevated blood glucose:  9.36  Group at risk of hypoglycaemia:  10.24 | Group with elevated blood glucose:  1.19  Group at risk of hypoglycaemia:  1.44 |
| Chaugule 2017a | MDI+CGM vs MDI+SMBG.  Base case treatment effects:  MDI+CGM reduced mean HbA1c by 1.0% (10.9mmol/mol) and severe hypoglycaemia event rates by 50% relative to MDI+SMBG. | CAD | 50 years | 339,196 (95% CI: 338,567–339,825) | 225,862 (95% CI: 225,278–226,447) | 113,334 (95% CI: 112,468–114,199) | 8.382 (95% CI: 8.375–8.388) | 5.027 (95% CI: 5.023–5.032) | 3.354 (95% CI: 3.346–3.326) |
| Chaugule 2017b | CGM+(CSII/MDI) vs SMBG+(CSII/MDI)  Base case treatment effects:  CGM+(CSII/MDI) reduced mean HbA1c by 0.6% (6.6mmol/mol) and the number of BSL tests per day from 8 to 2.8, when compared to SMBG+(CSII/MDI). | GBP | 4 years (budget impact analysis) | Costs: 10,770,671  Offsets: 8,116,912  Net impact:  2,653,760  (year 1)  Costs: 10,783,195  Offsets: 8,319,835  Net impact:  2,463,361  (year 2)  Costs: 11,317,177  Offsets: 8,527,830  Net impact:  3,402,143  (year 3)  Costs:  11,329,095  Offsets:  8,741,026  Net impact:  2,588,068  (year 4) | N/A | N/A | N/A | N/A | N/A |
| Heller 2017 | CSII+SMBG vs MDI+SMBG  Base case treatment effects:  Statistical models fitted to estimate effectiveness:  Beta regressions for HbA1c and negative binomial regressions for incidence of severe hypoglycaemia and DKA.  Both groups also underwent ‘dose adjustment for normal eating’ (DAFNE) training. | GBP | Lifetime (primary analysis)  Economic Evaluation Alongside Clinical Trials (2 years) | Deterministic:  90,581  Probabilistic: 100,617 | Deterministic:  70,132  Probabilistic:  81,785 | Deterministic: 20,448  Probabilistic:  18,832  2,959 (95% CI: 2,692 – 3,227) | Deterministic: 12.8166  Probabilistic:  13.1105 | Deterministic: 12.6719  Probabilistic:  12.9845 | Deterministic:  0.1447  Probabilistic:  0.1260  -0.004 (95% CI:  -0.057 – +0.048) |
| Jendle 2017 | Integrated (low glucose suspend) vs CSII+SMBG  Base case treatment effects:  Group at risk of hypoglycaemia: Integrated: 0 severe hypoglycaemic events per 100 patient-months.  CSII+SMBG: 2.2 severe hypoglycaemic events per 100 patient-months.  Equivalent HbA1c between groups was assumed.  Group with elevated blood glucose (HbA1c >8.0% [64mmol/mol]):  Integrated: HbA1c reduction of 0.58% (6.3mmol/mol)  CSII+SMBG: HbA1c reduction of 0.14% (1.5mmol/mol)  Equivalent rates of severe hypoglycaemia between groups was assumed (2.6 events per 100 patient-months).  Also assumed sensors were used 80% of the time. | SEK  EUR  SEK  EUR | Lifetime | Group at risk of hypoglycaemia: 2,671,858  279,969  Group with HbA1c >8.0% (64mmol/mol):  1,925,040  201,713 | Group at risk of hypoglycaemia: 2,409,462  252,473  Group with HbA1c >8.0% (64mmol/mol):  1,656,141  173,537 | Group at risk of hypoglycaemia: 262,396  27,495  Group with HbA1c >8.0% (64mmol/mol):  268,899  28,176 | Group at risk of hypoglycaemia: 13.110  Group with HbA1c >8.0% (64mmol/mol):  9.224 | Group at risk of hypoglycaemia: 11.233  Group with HbA1c >8.0% (64mmol/mol):  8.157 | Group at risk of hypoglycaemia: 1.877  Group with HbA1c >8.0% (64mmol/mol):  1.067 |
| Roze 2017 | Integrated (low glucose suspend) vs CSII+SMBG (assumed the modelled control group utilised SMBG).  Base case treatment effects:  Group with elevated glucose:  Integrated: Mean HbA1c change  -0.56% (6.1mmol/mol).  CSII+CGM: Mean HbA1c change  -0.13% (1.4mmol/mol).  Equivalent rates of severe hypoglycaemia was modelled between groups (2.6 events per 100 patient years).  Base case treatment effects:  Group at risk of hypoglycaemia:  Integrated: 0 events per 100 patient months.  CSII+CGM: 2.2 events per 100 patient months. | DKK | Lifetime | Group with elevated blood glucose:  2,027,316  Group at risk of hypoglycaemia:  2,277,868 | Group with elevated blood glucose: 1,801,293  Group at risk of hypoglycaemia:  2,109,186 | Group with elevated blood glucose:  226,023  Group at risk of hypoglycaemia:  168,682 | Group with elevated blood glucose:  12.44  Group at risk of hypoglycaemia:  13.08 | Group with elevated blood glucose:  10.99  Group at risk of hypoglycaemia:  11.20 | Group with elevated blood glucose:  1.45  Group at risk of hypoglycaemia:  1.88 |
| Bilir 2018 | Flash+(CSII/MDI) vs SMBG+(CSII/MDI)  Base case treatment effects:  Both groups increased HbA1c levels by 0.12(0.45)% [1.32 mmol/mol (4.95)] compared to baseline. Both groups had equivalent rates of severe hypoglycaemia (282.24 events per 100 person-years requiring non-medical assistance and 37.76 events per 100 person-years requiring medical assistance). The group using FGM had 4897.10 non severe hypoglycaemic events per 100 person-years compared to the SMBG with 6760.00 events per 100 person-years. The group using FGM had 25% of hypoglycaemic events at night compared to the SMBG group that had 27% of events at night. | SEK | 50 years | 1,222,333 | 989,051 | 233,283 | 13.26 | 12.46 | 0.801 |
| Conget 2018 | Integrated (low glucose suspend) vs CSII+SMBG  Base case treatment effects:  Integrated: 0 events of severe hypoglycaemia per 100 patient months.  CSII+SMBG: 2.2 events of severe hypoglycaemia per 100 patient months. | EUR | Lifetime | Direct costs: 112,445  Total costs: 217,272 | Direct costs: 64,780  Total costs: 176,236 | Direct costs: 47,665  Total costs: 41,036 | 13.11 | 11.23 | 1.88 |
| Garcia-Lorenzo 2018 | CGM+(CSII/MDI) vs SMBG+(CSII/MDI)  Base case treatment effects:  CGM+(CSII/MDI) reduced mean HbA1c by 0.23% (2.5mmol/mol) when compared to SMBG+(CSII/MDI). Equivalent rates of severe hypoglycaemia between groups was assumed. | EUR | Lifetime | 391,273 | 273,137 | 118,135 | 20.11 | 20.06 | 0.046 |
| Health Quality Ontario 2018 | MDI+SMBG  vs  MDI+CGM  Base case treatment effects:  Mean (95% CI) HbA1c change of  -0.57 (-0.78, -0.41)% [-6.2 (-8.5, -4.5)mmol/mol] favouring MDI+CGM.  MDI+SMBG  vs  CSII+CGM (Medtronic)  Base case treatment effects:  Mean (95% CI) HbA1c change of  -1.00 (-1.08, -0.92)% [-10.9 (-11.8, -10.1)mmol/mol] favouring CSII+CGM.  CSII+SMBG  vs  CSII+CGM (Dexcom)  Base case treatment effects:  Mean (95% CI) HbA1c change of  -1.09 (-1.37, -0.81)% [-11.9 (-15,  -8.9)mmol/mol] favouring CSII+CGM.  CSII+SMBG  vs  CSII+CGM (Medtronic)  Base case treatment effects:  Mean (95% CI) HbA1c change of  -0.68 (-1.04, -0.18)% [-7.4 (-11.4,  -2)mmol/mol] favouring CSII+CGM.  CGM+(CSII/MDI) vs  SMBG+(CSII/MDI)  Net budget impact (funding CGM for the entire population with type 1 diabetes).  Treatment effects:  Relative risk for severe hypoglycaemia in the group using CGM compared to SMBG was 0.869 (0.476, 1.586), and authors estimated the relative risk for each 1% reduction in mean HbA1c assuming a log-linear relationship. | CAD  CAD | Lifetime  5 years (budget impact analysis) | 229,413  258,306  257,947  258,373  Year 1  CGM: 481,949,914  SMBG: 166,77,4215  Impact: 315,175,698  Year 2  CGM: 527927021  SMBG: 206,074,531  Impact: 321,852,490  Year 3  CGM: 563,034,675  SMBG: 237,230,078  Impact: 325,804,597  Year 4  CGM: 590,099,986  SMBG: 262,185,663  Impact: 327,914,323  Year 5  CGM: 610,633,755  SMBG: 281,753,568  Impact: 328,880,187 | 125,586  125,586  177,320  177,320  N/A | 103,827  132,720  80,627  81,052  N/A | 18.906  18.944  18.916  18.949  N/A | 18.812  18.812  18.812  18.812  N/A | 0.094  0.132  0.104  0.137  N/A |
| Hellmund 2018 | FGM+(CSII/MDI) vs SMBG+(CSII/MDI)  Base case (for cost calculations):  FGM: Mean 0.5 BSL tests per day (in addition to utilising FGM).  SMBG: Mean of 10 BSL tests per day for those using SMBG. | GBP | 1 year | 970.23 | 1,204.50 | -234.27 | N/A | N/A | N/A |
| Herman 2018 | MDI+SMBG (1 BSL test per day) vs  MDI+SMBG  CSII+SMBG  CSII+CGM  Base case treatment effects:  Data from the Diabetes Control and Complications trial / epidemiology of diabetes interventions and complications (DCCT/ EDIC) trial for excellent (“~7.0%” [53mmol/mol]) vs poor glycaemic control (“~9.0%” [75mmol/mol]). | USD | 30 years | CSII+CGM:  442,420  CSII+SMBG:  256,400  MDI+SMBG:  213,925 | MDI+SMBG (1 BSL test per day): 154,432 | CSII+CGM:  231,833  CSII+SMBG:  45,812  MDI+SMBG:  3,337 | N/A | N/A | CSII+CGM:  0.87  CSII+SMBG:  0.87  MDI+SMBG:  0.87 |
| Nicolucci 2018 | Integrated (low glucose suspend) vs CSII+SMBG  Base case treatment effects:  Group at risk of hypoglycaemia: Integrated: 0 severe hypoglycaemic events per 100 patient-months.  CSII+SMBG: 2.2 severe hypoglycaemic events per 100 patient-months.  Equivalent HbA1c between groups was assumed.  Group with elevated blood glucose (HbA1c >8.0% [64mmol/mol]):  From reported values, we assumed authors modelled a relative reduction of HbA1c by 0.3% (3.3mmol/mol) in favour of the integrated system. | EUR | Lifetime | Group at risk of hypoglycaemia: 358,761  Group with HbA1c >8.0% [64mmol/mol]:  324,991 | Group at risk of hypoglycaemia: 295,521  Group with HbA1c >8.0% [64mmol/mol]:  259,852 | Group at risk of hypoglycaemia: 63,240  Group with HbA1c >8.0% [64mmol/mol]:  65,139 | Group at risk of hypoglycaemia: 13.081  Group with HbA1c >8.0% [64mmol/mol]:  12.442 | Group at risk of hypoglycaemia: 11.204  Group with HbA1c >8.0% [64mmol/mol]:  10.994 | Group at risk of hypoglycaemia: 1.877  Group with HbA1c >8.0% [64mmol/mol]:  1.448 |
| Pollard 2018 | CSII+SMBG vs MDI+SMBG  Base case treatment effects:  HbA1c (average annual progression):  +0.052% (0.6mmol/mol) for CSII+SMBG  +0.054% (0.6mmol/mol) for MDI+SMBG  Mean (95% CI) incident rate ratio comparing CSII+SMBG to MDI+SMBG at years one and two.  DKA: 1.40 (0.55, 3.58) in year 1 and 0.93 (0.23, 3.69) in year 2.  Severe hypoglycaemia:  1.33 (0.49, 3.65) in year 1 and 0.35 (0.08, 1.44) in year 2.  Both groups also underwent ‘dose adjustment for normal eating’ (DAFNE) training. | GBP | Lifetime | 99,337 | 80471 | 18,853 | 13.011 | 12.8785 | 0.1326 (95% CI:  -0.7087 – 0.9623) |
| Wan 2018 | MDI+CGM vs MDI+SMBG  Base case treatment effects:  For MDI+CGM, the mean (SD) HbA1c reduction was 0.60% (0.74) [6.6 mmol/mol (8.1)] greater, mean (SD) daily BSL tests were 0.55 (1.5) fewer, and the daily rate of non-severe hypoglycaemia was 0.07 lower than those utilising MDI+SMBG. | USD | Lifetime | 360,486 | 305,278 | 55,208 | 13.32 | 12.78 | 0.54 |
| Jendle 2019 | Hybrid closed loop vs CSII+SMBG  Base case treatment effects:  Group using hybrid closed loop systems: HbA1c reduced by 0.5% (5.5mmol/mol), from a baseline of 7.4% (57 mmol/mol) to 6.9% (52mmol/mol). No events of DKA or severe hypoglycaemia occurred while using hybrid closed loop systems.  Group using CSII+SMBG: It was assumed HbA1c would not change and that there would be no events of DKA. Severe hypoglycaemia occurred at a rate of 25 events per 100 person-years requiring medical assistance, and 65 events per 100 person-years requiring nonmedical assistance. | SEK | Lifetime | 3,345,882 | 3,034,327 | 311,555 | 15.35 | 13.45 | 1.90 |
| Roze 2019 | Low glucose suspend vs CSII+SMBG  Base case treatment effects:  Group with elevated blood glucose:  Mean reduction in HbA1c of 0.42% from low glucose suspend or 0.10% for CSII+SMBG therapy. Assumed equivalent rates of severe hypoglycaemia being 2.6 events per 100 patient years. Low glucose suspend comprised 43 sensors per year (4.95 days per week of sensor use).  Group at risk of hypoglycaemia:  Modelled 2.2 events of severe hypoglycaemia per 100 patient months among participants using CSII+SMBG, and zero events per 100 patient months among those using low glucose suspend enabled technology. | EUR | Lifetime | Group with elevated blood glucose:  189,855  Group at risk of hypoglycaemia:  204,013 | Group with elevated blood glucose:  150,366  Group at risk of hypoglycaemia:  171,032 | Group with elevated blood glucose:  39,489  Group at risk of hypoglycaemia:  32,981 | Group with elevated blood glucose:  15.54  Group at risk of hypoglycaemia:  16.7 | Group with elevated blood glucose:  13.77  Group at risk of hypoglycaemia:  14.53 | Group with elevated blood glucose:  1.77  Group at risk of hypoglycaemia:  2.16 |
