# Supplementary material for: Cost-effectiveness of health technologies in adults with type 1 diabetes: a systematic review and narrative synthesis
Source: Syst Rev. 2020 Aug 3;9:171. doi: 10.1186/s13643-020-01373-y (PMC7401226; doi:10.1186/s13643-020-01373-y)
Supplement: Supplementary file 4 — Additional file 4. Table 2. Study summaries. Description: Table outlining summaries of each study in the systematic review. [file 13643_2020_1373_MOESM4_ESM.docx]

| **Table 2. Study summaries.** | | | | | | | | |
| --- | --- | --- | --- | --- | --- | --- | --- | --- |
| **Study ID** | **Analysis and perspective** | **Modelled cohort (mean [SD])** | **Funding** | **Discount rate(s) /**  **Time horizon** | **Currency (year)** | **ICER (local currency) /**  **Willingness-to-pay threshold** | **ICER (2019 AUD)** | **ICER (2019 USD)** |
| Scuffham 2003 | Study author developed Markov model comparing CSII+SMBG with MDI+SMBG.  Perspective: Healthcare funder (the National Health Service in the UK). | N/A | Commercial: Medtronic (partly funded). | Health outcomes: 1.5%  Costs: 6.0%  Horizon: 8 years | GBP (2001) | 11,461 (3,656)  <30,000 (assumed / not reported)  If the threshold was 12,500: 70.1% of iterations / simulations were cost-effective.  If the threshold was 15,000: 81.4% of iterations / simulations were cost-effective. | 31,257 | 21,990 |
| Roze 2005 | Center for Outcomes Research & Evaluation (CORE) model comparing CSII+SMBG with MDI+SMBG.  Perspective: Healthcare funder (the National Health Service in the UK). | Age: 26 years  Diabetes duration: 12 years  HbA1c: 8.68% (71mmol/mol) | Commercial: Medtronic AG. | Health outcomes: 3.0%  Costs: 3.0%  Horizon: 60 years | GBP  (2003) | 25,648  <30,000 | 68,340 | 48,079 |
| Cohen 2007 | CORE model comparing CSII+SMBG with MDI+SMBG.  Perspective: Healthcare funder (the Australian healthcare system). | Age: 43.3 (16.2) years  Diabetes duration: 17.2 (12.5) years  HbA1c: 8.2 (1.8)%  [66 (19.7) mmol/mol]  Limited to adults. | Commercial: Medtronic Australasia. | Health outcomes: 5.0%  Costs: 5.0%  Horizon: 60 years | AUD (2006) | 74,147  <50,000 (assumed / not reported)  (Cost per life-year gained 88,220 with a threshold value of 76,000 per life year gained reported for the Australian context). | 100,287 | 70,541 |
| St Charles 2009a | CORE model comparing CSII+SMBG with MDI+SMBG.  Perspective: third-party payer (in the United States of America). | Age: 27.0 years  Diabetes duration: 9.0 years  HbA1c: 8.95% (74mmol/mol) | Commercial: Medtronic Diabetes. | Health outcomes: 3.0%  Costs: 3.0%  Horizon: 60 years | USD  (2007) | 16,992  <50,000 (93.8% of iterations/simulations were cost-effective). | 29,860 | 21,008 |
| St Charles 2009b | CORE model comparing CSII+SMBG with MDI+SMBG.  Perspective: Healthcare funder (the Canadian provincial government). | Age: 27.0 years  Diabetes duration: 9.0 years  HbA1c: 8.95% (74mmol/mol) | Commercial: Medtronic of Canada Ltd. | Health outcomes: 5.0%  Costs: 5.0%  Horizon: 60 years | CAD  (2006) | 23,797  <50,000 (assumed).  Results were presented for thresholds of: 30,000 (75% of iterations/simulations were cost-effective)  40,000 (91% of iterations/simulations were cost-effective)  50,000 (97% of iterations/simulations were cost-effective) | 31,547 | 22,194 |
| Cummins 2010 | CORE model comparing CSII+SMBG with MDI+SMBG.  Perspective: Healthcare funder (the National Health Service in the UK). | Age range: 20-39 years  HbA1c: 8.8% (73mmol/mol) | Non-commercial funding: Commissioned on behalf of the National Institute for Health and Clinical Excellence (NICE). | Health outcomes: Not reported  Costs: Not reported  Horizon: 50 years | GBP  (2006) | General population with type 1 diabetes: 37,712  Individuals with high risk of hypoglycaemia: 36,587  <20,000 or <30,000 | 95,412  91,356 | 67,125  64,271 |
| Huang 2010 | Study author developed Monte Carlo based  Markov simulation model comparing CGM+(CSII/MDI) with SMBG+(CSII/MDI).  Perspective: Societal (in the United States of America [USA]). | Cohort >25 years of age and HbA1c >7.0%.  Age: 43 (10) years.  Diabetes duration: “Age and gender specific”. | Non-commercial funding: Juvenile Diabetes Research Foundation (JDRF).  Commercial: Dexcom, Medtronic MiniMed, Abbott Diabetes Care, and LifeScan. | Health outcomes: 3.0%  Costs: 3.0%  Horizon: Lifetime | USD  (2008 was assumed because it was the latest year for any costing data. Inflation not reported). | 98,679  <100,000 | 165,846 | 116,677 |
| McQueen 2011 | Study author developed Markov model comparing CGM+(CSII/MDI) with SMBG+(CSII/MDI).  Perspective: Societal (in the USA). | Age: 40 years  Diabetes duration: 20 years  HbA1c: 7.6 (0.5)%  [60 (5.5) mmol/mol] | No funding. | Health outcomes: 3.0%  Costs: 3.0%  Horizon: 33 years | USD (2007) | 45,033  <100,000 (70% of iterations / simulations were cost-effective). | 79,161 | 55,692 |
| Kamble 2012 | CORE model comparing CSII+CGM to MDI+SMBG  Perspective: the USA health care system (societal perspective in addition). | Age: 41.2 (12.2) years.  Diabetes duration: 20.2 (11.9) years.  HbA1c: 8.3 (0.5)%  [67 (5.5) mmol/mol] | Non-commercial: Duke University.  Commercial: Medtronic. | Health outcomes: 3.0%  Costs: 3.0%  Horizon: 60 years | USD  (2010) | 229,675 (95% CI: 139,071–720,865)  <100,000 | 383,717 | 269,955 |
| Kamble 2013 | Patient time and costs from a clinical trial comparing CSII+CGM with MDI+SMBG. | Age:  Overall: 31.9 (17.0) years. Range 7-70 years.  CSII+CGM: 32.2 (17.5) years.  MDI+SMBG: 31.6 (16.5) years.  Diabetes duration: Overall: 15.4 (12.3) years  HbA1c: 8.3% (67mmol/mol)  Baseline diabetes care: four hours per week. | Commercial: Agreement between Duke University and Medtronic Diabetes. | Discount: N/A  Horizon: trial period (52 weeks). | USD (2010) | Patient time costs (per person per year) related to diabetes care (wages only):  4,600 (3,533) vs 3,523 (3,318)  Difference of 1,077 (95% CI: 491–1,638). P<0.001  Patient time costs (per person per year) related to diabetes care (wages plus fringe benefits):  6,612 (5,077) vs 5,064 (4,769)  Difference of 1,549 (95% CI: 705–2,354) | Difference:  1,800 (95% CI: 797–2,659)  (Adjusted to 2019 AUD).  Difference:  2,589 (95% CI: 1,144–3,821)  (Adjusted to 2019 AUD). | Difference: 1,266 (95% CI: 577–1,926)  (Adjusted to 2019 USD).  Difference:  1,821 (95% CI: 829–2,768)  (Adjusted to 2019 USD). |
| Ly 2014 | Trial based (stepped) economic evaluation comparing an integrated system (low glucose suspend) with CSII+SMBG.  Perspective: Healthcare funder (the Australian health care system). | Age: 18.6 (11.8) years. Range: 4–50 years.  Impaired awareness of hypoglycaemia. | Commercial: Medtronic.  Non-Commercial: JDRF. | Discount: N/A  Horizon: 6 months (trial duration) | AUD (2013) | ICER (cost per event-free year for all patients): 17,602  Cost per QALY gained (sample >12 years old): 40,803  < 50,000 (assumed / not reported) | 45,825 | 32,233 |
| NICE 2015 | IMS (Information Medical Statistics) CORE model comparing MDI+CGM with MDI+SMBG (at SMBG frequencies of 2, 4, 6, 8, and 10 times per day).  Perspective: Healthcare funder (the National Health System in the UK) and societal perspectives. | Age: 27 years.  Diabetes duration: 9.10 years.  HbA1c: 9.3% (78mmol/mol) | Non-commercial: National Institute for Health and Care Excellence. | Health outcomes: 3.5%  Costs: 3.5%  Horizon(s):  80 years. | GBP (2014) | Net monetary benefit calculated at a threshold of 20,000 (GBP) per QALY gained:  SMBG 2: 197,925  SMBG 4: 213,184  SMBG 6: 219,368  SMBG 8: 222,363  SMBG 10: 222,778  CGM: 160,928  ICER (deterministic):  CGM vs SMBG 2: 51,803  CGM vs SMBG 4: 192,982  CGM vs SMBG 6: -436,850  CGM vs SMBG 8: -157,980  CGM vs SMBG 10: -118,424  ICER (probabilistic):  CGM vs SMBG 2: 61,291  CGM vs SMBG 4: 234,164  CGM vs SMBG 6: -526,168  CGM vs SMBG 8: -156,537  CGM vs SMBG 10: -104,698  <20,000 – 30,000 | 104,745  390,208  -883,309  -319,434  -239,453  123,931  473,479  -1,063,910  -316,518  -211,698 | 73,691  274,522  -621,431  -224,730  -168,461  87,189  333,105  -748,489  -222,679  -148,935 |
| Roze 2015 | CORE model comparing CSII+CGM with CSII+SMBG.  Perspective: Societal payer perspective (in Sweden). | Age: 27 years.  Diabetes duration: 13 years.  HbA1c: 8.6% (70mmol/mol) | Commercial: Medtronic. | Health outcomes: 3.0%  Costs: 3.0%  Horizon: Lifetime | SEK (2011) | 367,571  <500,000 (>70% of iterations / simulations were cost-effective). | 61,129 | 43,003 |
| Bronstone 2016 | Study author cost-calculation comparing CGM+(CSII/MDI) with SMBG+(CSII/MDI). | Hypothetical health plan with 10 million adults.  Age: 18-64 years.  Estimated 46,500 with type 1 diabetes, and 9,300 of these with hypoglycaemia unawareness. | Commercial: Dexcom, Inc. | Discount: N/A  Horizon: 1 year | USD  (2014) | Cost saving (difference in costs for hospitalisation minus the cost of CGM): 8,799,000–12,519,000.  946–1,346 per patient | Cost saving:  13,548,676–19,276,722  1,457–2,073 per patient  (Adjusted to 2019 AUD). | Cost saving:  9,531,857–13,561,690  1,025–1,458 per patient  (Adjusted to 2019 USD). |
| Gomez 2016 | CORE model comparing integrated systems (CGM with alarms) with MDI+SMBG.  Perspective: Healthcare payer’s perspective (in Colombia). | Age: 34.19 (17.14) years  Diabetes duration: 13.96 (9.91) years  HbA1c: 9.0 (2.0)%  [75 (21.9) mmol/mol] | Commercial: Medtronic PLC. | Health outcomes: 5%  Costs: 5%  Horizon: 55 years | COP / USD  (2013) | COP  43,795,218 (total costs)  44,835,034 (direct costs)  USD  23,400 (total costs)  24,000 (direct costs)  Willingness-to-pay threshold:  Assumed to be $26,750 USD (approximately three times the gross domestic product per capita) per QALY | 43,066  43,694 | 30,298  30,740 |
| Haahtela 2016 | Real option analysis approach with cash flow simulation comparing integrated systems (hybrid closed loop) with MDI+SMBG.  Perspective: Market perspective (Finland). | N/A | Commercial: Not reported.  Non-commercial: Not reported. | Horizon: lifetime. | EUR  (2015 assumed) | Lifetime cost saving of 600,000 (ICERs not presented). | 1,000,792 (cost saving adjusted to 2019 AUD). | 704,029 (cost saving adjusted to 2019 USD). |
| Riemsma 2016 | CORE model comparing MiniMed Paradigm Veo and the Vibe with G4 Platinum CGM systems to:  CSII+CGM  CSII+SMBG  MDI+CGM  MDI+SMBG  Perspective: Healthcare funder (the National Health Service in England and Wales). | Age: 41.6 (12.8) years.  Diabetes duration: 27.1 (12.5) years.  HbA1c: 7.26 (0.71)%.  [56 (7.8) mmol/mol] | Non-commercial: The National Institute for Health Research Technology Assessment programme. | Health outcomes: 3.5%  Costs: 3.5%  Horizon: 80 years | GBP (2014) | Probabilistic:  **All technologies:**  CSII+SMBG: 52,381  CSII+CGM: 660,376  Veo extendedly dominated by stand-alone CSII+CGM  Vibe dominated by stand-alone CSII+CGM  **Intervention vs control**:  MiniMed Paradigm Veo vs:  MDI+SMBG 123,375  CSII+SMBG 730,501  CSII+CGM 422,849  Vibe with G4 Platinum CGM vs:  MDI+SMBG 133,323  CSII+SMBG 668,789  CSII+CGM Undefined  <30,000 | 105,915  1,334,637  249,465  1,477,075  855,031  269,579  1,350,689  Undefined | 74,514  938,952  175,505  1,039,160  601,536  189,656  950,245  Undefined |
| Roze 2016a | CORE model comparing an integrated system (low glucose suspend) with CSII+SMBG  Perspective: Healthcare payer (the National Health Service in the UK). Secondary analysis for the societal perspective. | Age: 27  Diabetes duration: 13  HbA1c 10% (86mmol/mol) | Commercial: Medtronic International Trading Sarl | Health outcomes: 1.5%  Costs: 3.5%  Horizon: Lifetime | GBP (2013) | 12,233 (95% CI: 12,225-12,351)  <20,000-30,000 | 25,090 | 17,652 |
| Roze 2016b | CORE model comparing an integrated system (low glucose suspend) with CSII+SMBG.  Perspective: Healthcare payer. Secondary analysis for the societal perspective in France. | Group with elevated blood glucose:  Age: 36 (13.6) years.  Diabetes duration: 17 (10.8) years.  HbA1c: 9.0 (0.9)%.  [75 (9.8) mmol/mol]  Group at risk of hypoglycaemia:  Age 18.6 (11) years.  Diabetes duration: 12.0 (8.9) years.  HbA1c: 7.5 (95% CI: 7.2, 7.9)%  [58 (95% CI: 55, 63) mmol/mol] | Commercial: Medtronic International Trading Sarl. | Health outcomes: 4.0%  Costs: 4.0%  Horizon: Lifetime | EUR (2014) | Group with elevated blood glucose:  30,163  <30,000 (80% of iterations / simulations were cost-effective).  <50,000 (100% of iterations / simulations were cost-effective).  Group at risk of hypoglycaemia:  22,005  <30,000 – 50,000 | 50,761  37,006 | 35,709  26,033 |
| Chaugule 2017a | CORE model comparing MDI+CGM vs MDI+SMBG.  Perspective: Assumed healthcare payer perspective (in Canada). | Age: 46 years.  Diabetes duration: 19 years.  HbA1c: 8.6 (0.7)%.  [70 (7.7) mmol/mol)] | Commercial: Dexcom, Inc. | Health outcomes: 1.5%  Costs: 1.5%  Horizon: 50 years | CAD  (2016) | 33,789 (95% CI: 33,558–34,079)  <50,000 | 37,470 | 26,361 |
| Chaugule 2017b | Study author developed economic budget impact model comparing CGM+(CSII/MDI) to SMBG+(CSII/MDI).  Perspective: Health system (health service utilisation in North West London Clinical Commissioning Groups). | All ages included.  HbA1c: difference 0.6% (6.6mmol/mol).  Other cohort features: Impaired awareness of hypoglycaemia. | Commercial: Dexcom, Inc. | Discount: N/A. Assumed cost increase 2.5% per year  Horizon: 4 years | GBP  (2016-2017 budgets) | Net budget impact in North West London Clinical Commissioning Groups: 2,653,760 (year 1)  2,588,068 (year 4)  Study authors concluded minimal budget impact. | 5,328,578  5,196,673 | 3,748,793  3,655,994 |
| Heller 2017 | The Sheffield Type 1 Diabetes Policy Model version 1.3 and the Economic Evaluation Alongside Clinical Trials (EEACT) comparing CSI+SMBG with MDI+SMBG.  Perspective: Healthcare funder (the National Health System in the UK) and the personal social services perspective. | Age: 40.4 (13.4) years.  Diabetes duration: 18.0 (12.5) years.  HbA1c: 9.1 (1.7)%  [76.0 (18.6) mmol/mol]  (Further seven subgroups with baseline HbA1c ranging from 7.5% (58mmol/mol) to 9.5% (80mmol/mol) and based on intention to treat or per protocol populations). | Commercial: “The insulin pumps were provided free of charge and unconditionally by Medtronic”    Non-commercial:   - The UK Health Technology Assessment Programme (project No 08/107/01). - The study was commissioned by the National Institute for Health Research (NIHR). - The Research and Development Programmes of the Department of Health for England and the Scottish Health and Social Care Directorates supported the costs of consumables. | Health outcomes: 3.5%  Costs: 3.5%  Horizon(s):  Modelling: Lifetime (primary analysis)  EEACT: 2 years | GBP (2013 – 14) | Lifetime horizon:  Deterministic: 141,312  Probabilistic:  149,483  Probability that CSII+SMBG and DAFNE is cost-effective at a threshold of 20,000 per QALY gained was 15.4%  EEACT:  “Dominated” | 289,908  303,541 | 203,958  213,549 |
| Jendle 2017 | CORE model comparing an integrated system (low glucose suspend) with CSII + SMBG.  Perspective: Societal (in Sweden). | Group at risk of hypoglycaemia:  Age:  Integrated: 17.4 (10.6) years  CSII+SMBG: 19.7 (12.9) years.  Diabetes duration: Integrated: 9.8 (7.4) years.  CSII+SMBG: 12.1 (10.0) years.  HbA1c  Integrated: 7.6% (60mmol/mol). CSII+SMBG: 7.4% (57mmol/mol)  Group with HbA1c >8.0% (64mmol/mol):  Age: 46 (17) years.  Diabetes duration: 24 (15) years.  HbA1c: 7.92% | Commercial: Medtronic. | Health outcomes: 3.0%  Costs: 3.0%  Horizon: Lifetime. | SEK and EUR  (2015) | Group at risk of hypoglycaemia:  139,795 SEK  <300,000  14,648 EU  <31,435  Group with HbA1c >8.0% (64mmol/mol):  251,896 SEK  <300,000  26,395 EUR  <31,435 | 22,674  25,064  40,875  45,183 | 15,951  17,632  28,755  31,785 |
| Roze 2017 | CORE model comparing integrated systems (low glucose suspend) with non-integrated CSII+SMBG (assumed that SMBG was modelled in the control group).  Perspective: Societal (in Denmark). | Group with elevated blood glucose:  Age: 27 years.  Diabetes duration: 13.2 years.  HbA1c 8.1% (65mmol/mol).  Group at risk of hypoglycaemia:  Age: 18.6 years.  Diabetes duration: 11 years.  HbA1c: 7.5% (58mmol/mol). | Commercial: Medtronic International Sarl. | Health outcomes: 3.0%  Costs: 3.0%  Horizon: Lifetime | DKK  (2015) | Group with elevated blood glucose: 156,082  <225,000 DKK (approximately equivalent to 30,000 EUR). 90% of iterations / simulations were cost-effective.  <375,000 DKK (approximately equivalent to 50,000 EUR). 99% of iterations / simulations were cost-effective.  Group at risk of hypoglycaemia:  89,868 DKK  <225,000 DKK (approximately equivalent to 30,000 EUR). 100% of iterations / simulations were cost-effective. | 34,983  19,695 | 24,611  14,266 |
| Bilir 2018 | CORE Diabetes Model comparing FGM +(CSII/MDI) with SMBG+(CSII/MDI)  Perspective: Swedish societal perspective was cited (only direct costs were presented). | Age: 43.7 (13.9) years.  Diabetes duration: 22.0 (12.0) years.  HbA1c: 6.78 (0.58)% [51 (6.3)mmol/mol] | Commercial: Abbott Diabetes Care. | Health outcomes: 3.0%  Costs: 3.0%  Horizon: 50 years (lifetime) | SEK (2016) | 291,130  <400,000 – 500,000 | 46,705 | 32,857 |
| Conget 2018 | CORE model comparing an integrated system (low glucose suspend) with CSII+SMBG.  Perspective: Healthcare funder (the Spanish National Health System) and societal perspectives. | Age: 18.6 (11) years.  Diabetes duration: 12 (8.9) years.  HbA1c: 7.5 (95% CI: 7.2 – 7.9)%  [58 (95% CI: 55, 63) mmol/mol] | Commercial: Medtronic | Health outcomes: 3.0%  Costs: 3.0%  Horizon: Lifetime | EUR  (2016) | 25,394 (National Health System perspective)  21,862 (Societal perspective)  <30,000 (Spain) | 42,896  36,930 | 30,176  25,979 |
| García-Lorenzo 2018 | Study author developed Markov model comparing CGM+(CSII/MDI) with SMBG+(CSII/MDI).  Perspective: Healthcare funder (the Spanish National Health Service). | Age: 26 years.  Diabetes duration: N/A.  HbA1c: weighted mean difference 0.23% (2.5mmol/mol). | Non-commercial: Commission by the Spanish Ministry of Health. | Health outcomes: 3.0%  Costs: 3.0%  Horizon: Lifetime | EUR  (2017) | 2,554,723  <20,000–25,000 | 4,261,481 | 2,997,832 |
| Health Quality Ontario 2018 | Study author developed (adapted) Markov model comparing:  MDI+SMBG  vs  MDI+CGM  MDI+SMBG  vs  CSII+CGM (Medtronic +/- low glucose suspend)  CSII+SMBG  vs  CSII+CGM (Dexcom / stand-alone devices)  CSII+SMBG  vs  CSII+CGM (Medtronic +/- low glucose suspend)  Study author developed model for budget impact analysis.  Perspective: Healthcare funder (the Ontario Ministry of Health and Long-Term Care). | Age: 27 years.  Diabetes duration: 6 (range 1-15) years.  HbA1c: 8.8% (73mmol/mol)  Forecast 0.31% annual prevalence increase, and estimated prevalence of hypoglycaemia unawareness (25%). | Non-commercial | Health outcomes: 1.5%  Costs: 1.5%  Horizon: Lifetime  Horizon: 5 years (starting 2018) | CAD  (2017)  CAD  (2017) | 1,108,812  1,007,909  778,687  592,206  <50,000  315,175,698 (year 1 budget impact)  328,880,187 (year 5 budget impact) | 1,224,807  1,114,930  859,671  656,037  349,492,502  364,689,157 | 861,686  784,384  604,803  461,540  245,877,726  256,568,996 |
| Hellmund 2018 | Per-patient cost calculation comparing FGM+(CSII/MDI) with SMBG+(CSII/MDI).  Perspective: Healthcare funder (the National Health Service in the UK). | Mean (SD; range)  Age:  FGM: 42.4 (13.1; range 18–71) years.  SMBG: 45.0 (14.6; range 20–80) years.  Diabetes duration:  FGM: 21 (10; range 5–47) years  SMBG: 23 (13; range 5–59) years.  HbA1c:  FGM: 6.8 (0.5; range 4.4–8.0)%  [51 (5.5; 25–64) mmol/mol]  SMBG: 6.8 (0.6; range 4.8–8.4)%  [51 (6.6; 29–68) mmol/mol] | Commercial: Abbot Diabetes Care. | Health outcomes: N/A  Costs: N/A  Horizon: 1 year | GBP  (2015-2016) | Per-patient cost calculation  234 (19%) lower for FGM | 474 (adjusted to 2019 AUD). | 333 (adjusted to 2019 USD). |
| Herman 2018 | Treatment simulations applied to existing patient outcome data from a longitudinal study.  MDI+SMBG (1 BSL test per day) vs  MDI+SMBG  CSII+SMBG  CSII+CGM  Perspective: Healthcare sector (in the United States of America). | Not reported.  From the DCCT/ EDIC study comparing intensive management to standard care:  Age: 27.2 (7.1) years vs 26.7 (7.1) years.  Diabetes duration: 5.8 (4.2) years vs 5.5 (4.1) years.  HbA1c: 9.1 (1.6)%  [76 (17.5) mmol/mol] | Non-commercial: National Institute of Diabetes and Digestive and Kidney Disease, the National Eye Institute, National Institute of Neurologic Disorders and Stroke General Clinical Research Centres, and Clinical Translational Science Centre Program. | Health outcomes: 3.0%  Costs: 3.0%  Horizon: 30 years | USD  (2014) | MDI+SMBG: 3,835  CSII+SMBG: 52,654  CSII+CGM: 266, 457  <100,000 | 5,906  81,082  410,317 | 4,155  57,043  288,669 |
| Nicolucci 2018 | CORE model comparing an integrated system (low glucose suspend) with CSII + SMBG.    Perspective: Societal (in Italy). | Group at risk of hypoglycaemia: Age:  Integrated: 19.7 (12.9) years.  CSII+SMBG: 17.4 (10.6) years.  Diabetes duration:  Integrated: 12.1 (10.0) years.  CSII+SMBG: 9.8 (7.4) years  HbA1c:  Integrated: 7.4 (95% CI: 7.2, 7.6)%.  [57 (95% CI: 55, 60) mmol/mol]  CSII+SMBG: 7.6 (95% CI: 7.4, 7.9)%.  [60 (95% CI: 57, 63)mmol/mol]  Group with HbA1c >8.0% (64mmol/mol):  Age: 27 (15.6) years.  Diabetes duration: 13.2 (10.8) years. HbA1c: 8.1 (1.3)%  [65 (14.2) mmol/mol] | Commercial: Medtronic Italy. | Health outcomes: 3.0%  Costs: 3.0%  Horizon: lifetime | EUR  (2016) | Group at risk of hypoglycaemia:  44,982  Group with HbA1c >8.0% (64mmol/mol):  33,692  <60,000–80,000 | 56,191  75,025 | 39,528  52,778 |
| Pollard 2018 | The Sheffield Type 1 Diabetes Policy Model version 1.3.2, comparing CSII+SMBG with MDI+SMBG.  Perspective: Healthcare funder and personal social services (the National Health Service in the UK). | Age: 40.4 (13.3) years.  Diabetes duration 18.0 (12.3) years.  HbA1c: 9.1 (1.7)%  [76 (18.6) mmol/mol] | Commercial: Medtronic.  Non-Commercial: the UK Health Technology Assessment programme. | Health outcomes: 3.5%.  Costs: 3.5%  Horizon: Lifetime | GBP  (2013 / 2014) | 142,195  <20,000 or <30,000 | 291,685 | 205,208 |
| Wan 2018 | Modified Sheffield type 1 diabetes policy model comparing MDI+CGM with MDI+SMBG.  Perspective: Societal (in the United States of America). | Age:  CGM: 45.7 (13.6) years.  SMBG: 51.4 (10.9) years.  Diabetes duration:  CGM: 19.6 (13.1) years.  SMBG: 23.1 (14.5) years.  HbA1c:  CGM: 8.6 (0.7)%.  [70 (7.7) mmol/mol]  SMBG: 8.6 (0.6)%.  [70 (6.6) mmol/mol] | Commercial: Dexcom.  Non-commercial: the National Institute of Diabetes and Digestive and Kidney Diseases Chicago Center for Diabetes Translation Research. | Health outcomes: 3.0%  Costs: 3.0%  Horizon: Lifetime | USD  (2015) | 98,108 (95% CI: 90,298–105,144)  <100,000 | 157,234 | 110,619 |
| Jendle 2019 | CORE Diabetes Model comparing a hybrid closed loop system to CSII+SMBG.  Perspective: Societal (Sweden) | Age: 37.8 (16.5) years.  Diabetes duration: 21.7 years.  HbA1c: 7.4(0.9)% [57 (9.8) mmol/mol]. | Commercial: Medtronic International Trading Sàrl. | Health outcomes: 3.0%  Costs: 3.0%  Horizon: Lifetime | SEK (2018) | 164,236  <300,000: 97.6% of iterations / simulations were cost-effective. | 25,327 | 17,817 |
| Roze 2019 | CORE Diabetes Model comparing integrated system (low glucose suspend) with CSII+SMBG.  Perspective: Societal (the Netherlands). | Group with elevated blood glucose:  Age: 27 (15.6) years.  Diabetes duration: 13.2 (10.8) years.  HbA1c: 8.0%  (64 mmol/mol).  Group at risk of hypoglycaemia:  Age 18.6 (11.8) years.  Diabetes duration: 11.0 (8.9) years.  HbA1c: 7.5%  (58 mmol/mol). | Commercial: Medtronic International Trading Sàrl. | Health outcomes: 1.5%  Costs: 4.0%  Horizon: Lifetime | EUR (2014) | Group with elevated blood glucose:  22,325  <30,000: 88.5% of iterations / simulations were cost-effective.  Group at risk of hypoglycaemia:  15,243  <30,000: 99.5% of iterations / simulations were cost-effective. | 37,440  25,624 | 26,338  18,026 |
