# Supplementary material for: Cost-effectiveness of health technologies in adults with type 1 diabetes: a systematic review and narrative synthesis
Source: Syst Rev. 2020 Aug 3;9:171. doi: 10.1186/s13643-020-01373-y (PMC7401226; doi:10.1186/s13643-020-01373-y)
Supplement: Supplementary file 5 — Additional file 5. Table 3. Assessment of study relevance and credibility. Description: Table outlining the assessment of relevance and credibility for each study in the systematic review. [file 13643_2020_1373_MOESM5_ESM.docx]

| **Table 3. Assessment of study relevance and credibility.** | | |
| --- | --- | --- |
| **Study ID** | **Relevance** | **Credibility** |
| Scuffham 2003 | **Relevance**: Sufficient. | **Credibility**:  External validation: Neutral.  Internal verification: Neutral.  Face validity: Neutral.  Design: Neutral.  Data: Neutral.  Analysis: Strength.  Uncertainty: Strength.  Reporting: Strength.  Interpretation: Neutral.  Potential conflict of interest (COI): Weakness.  Addressing COI: Neutral. |
| Roze 2005 | **Relevance**: Sufficient. | **Credibility**:  External validation: Strength.  Internal verification: Strength.  Face validity: Strength.  Design: Strength.  Data: Strength.  Analysis: Strength.  Uncertainty: Strength.  Reporting: Strength.  Interpretation: Strength.  Potential COI: Weakness.  Addressing COI: Neutral. |
| Cohen 2007 | **Relevance**: Sufficient. | **Credibility**:  External validation: Strength.  Internal verification: Strength.  Face validity: Strength.  Design: Strength.  Data: Strength.  Analysis: Strength.  Uncertainty: Strength.  Reporting: Strength.  Interpretation: Neutral.  Potential COI: Weakness.  Addressing COI: Neutral. |
| St Charles 2009a | **Relevance**: Sufficient. | **Credibility**:  External validation: Strength.  Internal verification: Strength.  Face validity: Strength.  Design: Strength.  Data: Strength.  Analysis: Strength.  Uncertainty: Strength.  Reporting: Strength.  Interpretation: Strength.  Potential COI: Weakness.  Addressing COI: Neutral. |
| St Charles 2009b | **Relevance**: Sufficient. | **Credibility**:  External validation: Strength.  Internal verification: Strength.  Face validity: Strength.  Design: Strength.  Data: Strength.  Analysis: Strength.  Uncertainty: Strength.  Reporting: Strength.  Interpretation: Strength.  Potential COI: Weakness.  Addressing COI: Neutral. |
| Cummins 2010 | **Relevance**: Sufficient. | **Credibility**:  External validation: Strength.  Internal verification: Strength.  Face validity: Strength.  Design: Strength.  Data: Strength.  Analysis: Strength.  Uncertainty: Strength.  Reporting: Strength.  Interpretation: Strength.  Potential COI: Strength.  Addressing COI: N/A. |
| Huang 2010 | **Relevance**: Sufficient. | **Credibility**:  External validation: Neutral.  Internal verification: Neutral.  Face validity: Strength.  Design: Neutral.  Data: Neutral.  Analysis: Neutral.  Uncertainty: Neutral.  Reporting: Neutral.  Interpretation: Neutral.  Potential COI: Weakness.  Addressing COI: Strength. |
| McQueen 2011 | **Relevance**: Sufficient. | **Credibility**:  External validation: Neutral.  Internal verification: Neutral.  Face validity: Neutral.  Design: Neutral.  Data: Strength.  Analysis: Strength.  Uncertainty: Strength.  Reporting: Strength.  Interpretation: Strength.  Potential COI: Strength.  Addressing COI: N/A. |
| Kamble 2012 | **Relevance**: Sufficient. | **Credibility**:  External validation: Strength.  Internal verification: Strength.  Face validity: Strength.  Design: Strength.  Data: Strength.  Analysis: Strength.  Uncertainty: Strength.  Reporting: Strength.  Interpretation: Strength.  Potential COI: Weakness.  Addressing COI: Neutral. |
| Kamble 2013 | **Relevance**: Sufficient. | **Credibility**:  External validation: Neutral.  Internal verification: Neutral.  Face validity: Neutral.  Design: Neutral.  Data: Neutral.  Analysis: Neutral.  Uncertainty: Weakness.  Reporting: Neutral.  Interpretation: Neutral.  Potential COI: Weakness.  Addressing COI: Strength. |
| Ly 2014 | **Relevance**: Sufficient. | **Credibility**:  External validation: Neutral.  Internal verification: Neutral.  Face validity: Strength.  Design: Strength.  Data: Strength.  Analysis: Strength.  Uncertainty: Neutral.  Reporting: Strength.  Interpretation: Strength.  Potential COI: Weakness.  Addressing COI: Strength. |
| NICE 2015 | **Relevance**: Sufficient. | **Credibility**:  External validation: Strength.  Internal verification: Strength.  Face validity: Strength.  Design: Strength.  Data: Strength.  Analysis: Strength.  Uncertainty: Strength.  Reporting: Strength.  Interpretation: Strength.  Potential COI: Strength.  Addressing COI: N/A. |
| Roze 2015 | **Relevance**: Sufficient. | **Credibility**:  External validation: Strength.  Internal verification: Strength.  Face validity: Strength.  Design: Strength.  Data: Strength.  Analysis: Strength.  Uncertainty: Strength.  Reporting: Strength.  Interpretation: Strength.  Potential COI: Weakness.  Addressing COI: Neutral. |
| Bronstone 2016 | **Relevance**: Sufficient. | **Credibility**:  External validation: Neutral.  Internal verification: Neutral.  Face validity: Neutral.  Design: Neutral.  Data: Neutral.  Analysis: Strength.  Uncertainty: Neutral.  Reporting: Neutral.  Interpretation: Neutral.  Potential COI: Weakness.  Addressing COI: Neutral. |
| Gomez 2016 | **Relevance**: Sufficient. | **Credibility**:  External validation: Neutral.  Internal verification: Neutral.  Face validity: Neutral.  Design: Neutral.  Data: Neutral.  Analysis: Neutral.  Uncertainty: Neutral.  Reporting: Neutral.  Interpretation: Neutral.  Potential COI: Weakness.  Addressing COI: Neutral. |
| Haahtela 2016 | **Relevance**: Sufficient. | **Credibility**:  External validation: Neutral.  Internal verification: Neutral.  Face validity: Weakness  Design: Neutral.  Data: Weakness  Analysis: Neutral.  Uncertainty: Neutral.  Reporting: Neutral.  Interpretation: Neutral.  Potential COI: Neutral.  Addressing COI: Neutral. |
| Riemsma 2016 | **Relevance**: Sufficient. | **Credibility**:  External validation: Strength.  Internal verification: Strength.  Face validity: Strength.  Design: Strength.  Data: Strength.  Analysis: Strength.  Uncertainty: Strength.  Reporting: Strength.  Interpretation: Strength.  Potential COI: Strength.  Addressing COI: N/A. |
| Roze 2016a | **Relevance**: Sufficient. | **Credibility**:  External validation: Strength.  Internal verification: Strength.  Face validity: Strength.  Design: Strength.  Data: Strength.  Analysis: Strength.  Uncertainty: Strength.  Reporting: Strength.  Interpretation: Strength.  Potential COI: Weakness.  Addressing COI: Neutral. |
| Roze 2016b | **Relevance**: Sufficient. | **Credibility**:  External validation: Strength.  Internal verification: Strength.  Face validity: Strength.  Design: Strength.  Data: Strength.  Analysis: Strength.  Uncertainty: Strength.  Reporting: Strength.  Interpretation: Strength.  Potential COI: Weakness.  Addressing COI: Neutral. |
| Chaugule 2017a | **Relevance**: Sufficient. | **Credibility**:  External validation: Strength.  Internal verification: Strength.  Face validity: Strength.  Design: Strength.  Data: Strength.  Analysis: Strength.  Uncertainty: Strength.  Reporting: Strength.  Interpretation: Strength.  Potential COI: Weakness.  Addressing COI: Neutral. |
| Chaugule 2017b | **Relevance**: Sufficient. | **Credibility**:  External validation: Neutral.  Internal verification: Neutral.  Face validity: Strength.  Design: Strength.  Data: Strength.  Analysis: Strength.  Uncertainty: Neutral.  Reporting: Neutral.  Interpretation: Strength.  Potential COI: Weakness.  Addressing COI: Neutral. |
| Heller 2017 | **Relevance**: Sufficient. | **Credibility**:  External validation: Strength.  Internal verification: Strength.  Face validity: Strength.  Design: Strength.  Data: Strength.  Analysis: Strength.  Uncertainty: Strength.  Reporting: Strength.  Interpretation: Strength.  Potential COI: Weakness.  Addressing COI: Neutral. |
| Jendle 2017 | **Relevance**: Sufficient. | **Credibility**:  External validation: Strength.  Internal verification: Strength.  Face validity: Strength.  Design: Strength.  Data: Neutral.  Analysis: Strength.  Uncertainty: Strength.  Reporting: Strength.  Interpretation: Strength.  Potential COI: Weakness.  Addressing COI: Neutral. |
| Roze 2017 | **Relevance**: Sufficient. | **Credibility**:  External validation: Strength.  Internal verification: Strength.  Face validity: Strength.  Design: Strength.  Data: Strength.  Analysis: Strength.  Uncertainty: Strength.  Reporting: Strength.  Interpretation: Strength.  Potential COI: Weakness.  Addressing COI: Neutral. |
| Bilir 2018 | **Relevance**: Sufficient. | **Credibility**:  External validation: Strength.  Internal verification: Strength.  Face validity: Strength.  Design: Strength.  Data: Strength.  Analysis: Strength.  Uncertainty: Strength.  Reporting: Strength.  Interpretation: Strength.  Potential COI: Weakness.  Addressing COI: Neutral. |
| Conget 2018 | **Relevance**: Sufficient. | **Credibility**:  External validation: Strength.  Internal verification: Strength.  Face validity: Strength.  Design: Strength.  Data: Neutral.  Analysis: Strength.  Uncertainty: Strength.  Reporting: Strength.  Interpretation: Neutral.  Potential COI: Weakness.  Addressing COI: Neutral. |
| García-Lorenzo 2018 | **Relevance**: Sufficient. | **Credibility**:  External validation: Neutral.  Internal verification: Neutral.  Face validity: Neutral.  Design: Neutral.  Data: Neutral.  Analysis: Neutral.  Uncertainty: Strength.  Reporting: Neutral.  Interpretation: Strength.  Potential COI: Strength.  Addressing COI: N/A. |
| Health Quality Ontario 2018 | **Relevance**: Sufficient. | **Credibility**:  External validation: Neutral.  Internal verification: Neutral.  Face validity: Strength.  Design: Strength.  Data: Strength.  Analysis: Strength.  Uncertainty: Strength.  Reporting: Strength.  Interpretation: Strength.  Potential COI: Neutral.  Addressing COI: Neutral. |
| Hellmund 2018 | **Relevance**: Sufficient. | **Credibility**:  External validation: Neutral.  Internal verification: Neutral.  Face validity: Neutral.  Design: Neutral.  Data: Strength.  Analysis: Strength.  Uncertainty: Neutral.  Reporting: Strength.  Interpretation: Neutral.  Potential COI: Weakness.  Addressing COI: Neutral. |
| Herman 2018 | **Relevance**: Sufficient. | **Credibility**:  External validation: Neutral.  Internal verification: Neutral.  Face validity: Strength.  Design: Strength.  Data: Neutral.  Analysis: Strength.  Uncertainty: Neutral.  Reporting: Strength.  Interpretation: Strength.  Potential COI: Strength.  Addressing COI: N/A. |
| Nicolucci 2018 | **Relevance**: Sufficient. | **Credibility**:  External validation: Strength.  Internal verification: Strength.  Face validity: Strength.  Design: Strength.  Data: Neutral.  Analysis: Strength.  Uncertainty: Strength.  Reporting: Strength.  Interpretation: Strength.  Potential COI: Weakness.  Addressing COI: Neutral. |
| Pollard 2018 | **Relevance**: Sufficient. | **Credibility**:  External validation: Neutral.  Internal verification: Strength.  Face validity: Strength.  Design: Strength.  Data: Neutral.  Analysis: Neutral.  Uncertainty: Strength.  Reporting: Strength.  Interpretation: Strength.  Potential COI: Neutral.  Addressing COI: Strength. |
| Wan 2018 | **Relevance**: Sufficient. | **Credibility**:  External validation: Neutral.  Internal verification: Neutral.  Face validity: Strength.  Design: Strength.  Data: Strength.  Analysis: Strength.  Uncertainty: Strength.  Reporting: Strength.  Interpretation: Strength.  Potential COI: Weakness.  Addressing COI: Neutral. |
| Jendle 2019 | **Relevance**: Sufficient. | **Credibility**:  External validation: Strength.  Internal verification: Strength.  Face validity: Strength.  Design: Strength.  Data: Strength.  Analysis: Strength.  Uncertainty: Strength.  Reporting: Strength.  Interpretation: Strength.  Potential COI: Weakness.  Addressing COI: Neutral. |
| Roze 2019 | **Relevance**: Sufficient. | **Credibility**:  External validation: Strength.  Internal verification: Strength.  Face validity: Strength.  Design: Strength.  Data: Strength.  Analysis: Strength.  Uncertainty: Strength.  Reporting: Strength.  Interpretation: Strength.  Potential COI: Weakness.  Addressing COI: Neutral. |
